# Evidence and evolutionary analysis of ancient whole-genome duplication in barley predating the divergence from rice

**DOI:** 10.1186/1471-2148-9-209

**Published:** 2009-08-22

**Authors:** Thomas Thiel, Andreas Graner, Robbie Waugh, Ivo Grosse, Timothy J Close, Nils Stein

**Affiliations:** 1 IPK Gatersleben, Corrensstr. 3, 06466 Gatersleben, Germany; 2 Scottish Crop Research Institute (SCRI), Invergowrie, Dundee DD2 5DA, UK; 3 Institut für Informatik, Martin-Luther-Universität Halle-Wittenberg, Von-Seckendorff-Platz 1, 06120 Halle (Saale), Germany; 4 Department of Botany and Plant Sciences, University of California, Riverside, CA, 92521-0124, USA

## Abstract

**Background:**

Well preserved genomic colinearity among agronomically important grass species such as rice, maize, Sorghum, wheat and barley provides access to whole-genome structure information even in species lacking a reference genome sequence. We investigated footprints of whole-genome duplication (WGD) in barley that shaped the cereal ancestor genome by analyzing shared synteny with rice using a ~2000 gene-based barley genetic map and the rice genome reference sequence.

**Results:**

Based on a recent annotation of the rice genome, we reviewed the WGD in rice and identified 24 pairs of duplicated genomic segments involving 70% of the rice genome. Using 968 putative orthologous gene pairs, synteny covered 89% of the barley genetic map and 63% of the rice genome. We found strong evidence for seven shared segmental genome duplications, corresponding to more than 50% of the segmental genome duplications previously determined in rice. Analysis of synonymous substitution rates (Ks) suggested that shared duplications originated before the divergence of these two species. While major genome rearrangements affected the ancestral genome of both species, small paracentric inversions were found to be species specific.

**Conclusion:**

We provide a thorough analysis of comparative genome evolution between barley and rice. A barley genetic map of approximately 2000 non-redundant EST sequences provided sufficient density to allow a detailed view of shared synteny with the rice genome. Using an indirect approach that included the localization of WGD-derived duplicated genome segments in the rice genome, we determined the current extent of shared WGD-derived genome duplications that occurred prior to species divergence.

## Background

Whole-genome duplications provide the genetic material for evolutionary innovation. Thinking about the evolutionary force behind the emergence of gene duplications goes back to Ohno [1], who stated that "natural selection merely modified while redundancy created". Since then, comparisons between whole-genome sequences have provided incontrovertible evidence that several plant genomes have undergone multiple ancient polyploidization events during their evolution. These include *Arabidopsis thaliana *[2], poplar [3], grape [4] and rice [5]. While polyploidization is generally followed by massive gene silencing and elimination of duplicated genes [6], the evolutionary novelty arises by the selective retention of duplicated genes across gene classes [7] leading to specialization [8] and changes in gene expression among gene pairs [9].

Today, it is commonly accepted that segmental gene duplications present within the rice genome originated from a single whole-genome duplication event (WGD) in a common ancestor at around 20 million years before the divergence of many cereal crops, including rice, Sorghum, the Triticeae and maize [10]. While the different numbers of 9, 10 or 18 duplicated blocks in the rice genome suggested by three WGD studies in rice [10-12] depend on the chosen analytical approach or the criteria that define duplicated blocks, actual differences do exist in the extent of these duplicated segments among rice chromosomes. For example, a single duplicated block between Os11 and Os12 involving together 9.71 Mb of the rice genome was identified in the third study [12], while two duplicated segments covering 23.5 Mb over both arms of the same chromosomes were found in the second study [11]. Divergent conclusions have also been reached concerning which rice chromosomes were involved in genome duplications. The third study [12] failed to detect the duplications between rice Os03 and Os12 as well as between Os04 and Os08 that were detected by both other studies. Precise delimitation of individual duplications at base pair-level was only considered in a single study [11] based on the assembly of the *Oryza sativa *ssp. *indica *whole genome shotgun sequence information. This dataset, however, showed considerable differences in the position of corresponding genes when compared to the *Oryza sativa *ssp. *japonica *assembly, mainly because of differences in the intergenic regions contributing to 28% misalignment rate between both genomes [11].

Apart from these ambiguities, the nearly complete genomic sequence for the *japonica *rice genome and the increased availability of genomic resources for other grass species now enables a more detailed understanding of the evolution of grass genomes. While initial studies based on the comparison of RFLP maps uncovered colinear relationship of genes between rice and other grass species [13,14], detail was generally limited by the paucity of mapped, sequence-based markers. The development of EST-derived genetic markers and subsequent saturation of genetic maps has both extended and refined the 'grass genome model' including the relationship between rice and barley [15,16]. Given the observed conservation of synteny among rice and the Triticeae, the existence of shared genome duplications has been reported in a number of studies [16-19].

Taking advantage of a comprehensive genetic map of barley comprising 1930 EST-derived markers and the availability of a well annotated and curated sequence of the genome of *Oryza sativa *ssp. *japonica *rice, the objectives of this study were (i) to revise and refine the rice WGD event, which is shared by all members of the grass family, (ii) to review barley-rice colinearity using an expanded set of mapped barley genes, and (iii) to detect, locate and analyze genome-wide remnants of the WGD in the barley genome.

## Results

### Fine-scale analysis of the rice whole-genome duplication

The availability of the complete genomic sequence of rice (*Oryza sativa *ssp. *japonica*) provides a reference dataset to study the WGD event shared by all cereals [10] as it provides the most detailed foot-print of the WGD that shaped the ancestral grass genome. Relating an increased understanding of the barley genome to that of rice should therefore enable us to identify shared duplicated segments that originate from their last common ancestor and that remain in the barley genome.

Based on release 5 (2007) of the *japonica *rice annotation consisting of 66710 gene models from 56278 predicted genes, we performed an all-versus-all sequence comparison (BlastN, bit score ≥ 100). Bearing in mind that ongoing local gene duplications in rice are common [20], several filtering steps were applied in order to identify rice gene pairs that originated from the WGD. Local gene duplications can potentially affect our ability to determinate precisely both the WGD in the rice genome sequence and barley – rice genome colinearity. They appear either as local tandem copies or, presumably as a result of undefined translocation events, randomly distributed throughout the rice genome where they form a 'background' of homologous genes [11]. We observed that 82% of the identified homologs of a query gene that were found on the same rice chromosome were located at a maximum distance of 1 Mb. This was taken as a strong indication that they originated from local gene duplication. Using 1 Mb as a cutoff, we identified 7147 locally duplicated rice genes (13% of all genes), spread ubiquitously across all rice chromosomes, but excluding the centromeric regions (Figure S1 of Additional file 1). In order to distinguish these 'background' duplications from those originating from the WGD, we (i) *a priori *excluded genes and their homologs from the analysis whenever they occurred at more than 5 distinct places (> 1 Mb) in the rice genome sequence and (ii) determined whether duplicated genes were arranged colinearly within any corresponding sister segments by applying three quality parameters: gene pair density weight > 2, maximum distance between two neighboring gene pairs = 500 kb, minimum number of gene pairs = 5 (see Methods for details). Statistical significance of observed segmental duplications was tested subsequently by the one-sided Fisher's exact test (Table S1 of Additional file 1) and the assignment step was cross-checked by inspecting dot-plots (Figure S2 of Additional file 1). Background duplications accounted for 11686 gene homologs in rice. Similar pairwise synonymous substitution rate distributions for gene pairs classified as local duplications (Ks median 0.37 from 2433 gene pairs) and background duplications (Ks median 0.45 from 5267 gene pairs) suggested a common time of origin and likely similar evolutionary mechanisms for both types.

**Figure 1:**
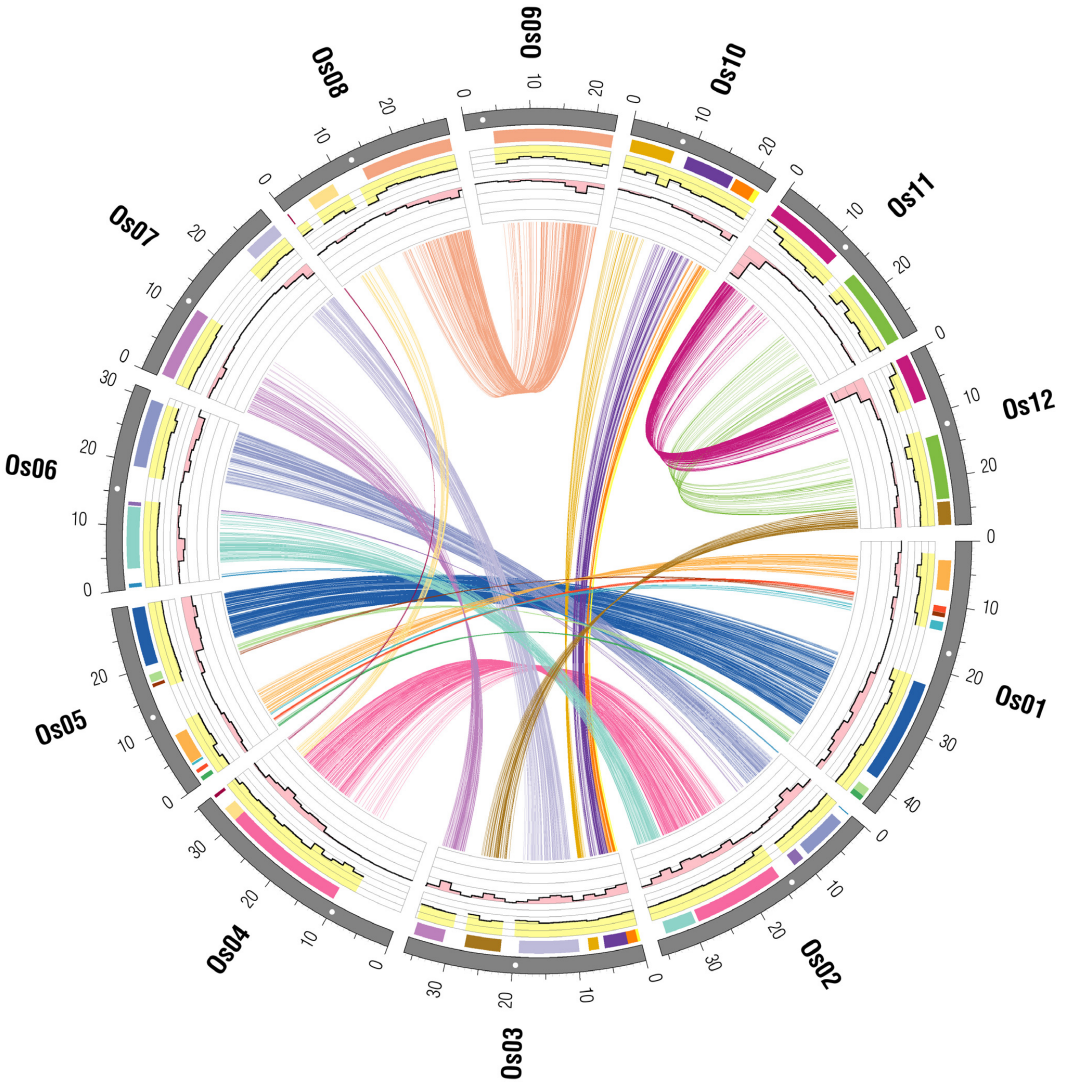
**Segmental duplications in the rice genome**. Segmentally duplicated rice genes are displayed by connecting lines among the 12 rice chromosomes (Os01 – Os12). Duplicated genes were organized in 24 pairs of genome segments located throughout the whole genome as indicated by differing colors of bars in the outer lane. Centromere positions are indicated by white dots. Mean Ks values of duplicated genes are shown in yellow bar plots along the 12 rice chromosomes (2 Mb bins, scale: 0 – 2, scale spacing 0.5). Histograms in pink show the ratio of duplicated genes to the total number of annotated genes (2 Mb bins, scale: 0 – 0.8, scale spacing: 0.2). Positions are in Mb.

After applying the above filtering steps, 4492 (8.0%) genes were determined as being segmentally duplicated genes and were organized in almost perfect colinearity between their respective rice chromosomes. They could be assigned to 24 pairs of genomic segments covering 261 Mb (70%) of the rice genome (Figure 1, Table S1 of Additional file 1). Duplicated segments were distributed across all 12 chromosomes, except their centromeric regions. This is opposed to the distribution of transposable element-related (TE-related) genes, which were preferably located at the centromeres (for the distribution of all rice genes in comparison to all non TE-related genes see Figure S1 of Additional file 1). Duplicated segments involved 57% (Os07) to 85% (Os11) of their respective chromosome sizes (Table S2 of Additional file 1). The overall genomic regions for which traces of WGD could be detected included 39582 annotated rice genes (including genes identified as TE-related genes by the Rice Genome Annotation group) or 70% of the rice transcriptome. That implies that for 89% of the genes located within such duplicated regions a corresponding paralog could not be detected. The ratio of segmentally duplicated to the total number of genes observed among duplicated chromosomal segments ranged from 2.6% (for segment os11_12_2) to 23% (os11_12_1), and varied within sub-domains of individual duplicated blocks (e.g. 5.9% versus 13% for os03_07_2, see Table S1 of Additional file 1). To examine position-specific patterns in the frequency ratio of segmentally duplicated versus total number of genes in more detail, the ratios were computed for physical bins of 2 Mb. Typically, higher ratios were found in the terminal bins (up to 20%) dropping towards the centromeres (down to less than 3%, e.g. for os02_04_1 and os08_09_1, see Figure 1). It should be noted that WGD-derived paralog retention ratios are possibly underestimated due to the emergence of additional gene copies through local gene duplications or transposition events that occurred after the WGD in rice and presumably affected all duplicated segments without bias (see Figure S1 of Additional file 1).

Segment pair os11_12_1 located on rice chromosomes Os11 (0.0 – 12.0 Mb) and Os12 (0.0 – 7.5 Mb) differed from all other duplicated segments by having higher ratios of 19% and 30% duplicated genes, respectively. A significantly lower mean Ks of 0.35 was characteristic of this segment pair compared to all other nine duplicated blocks that exceeded a size of 10 Mb and suggested a more recent origin (Tukey HSD test on the pairwise Ks means, p < 0.05 in all cases). More specifically, 60% (147/244) of all gene pairs in this region had Ks ratios smaller than 0.2 resulting in a right-skewed Ks distribution (median of 0.13). The 2 Mb-bin analysis showed that the first 2 Mb-bin of chromosomes 11 and 12 contributed significantly to the Ks ratio difference observed for the other WGD derived chromosomal segment pairs. Here, the ratio of duplicated genes was as high as 65% and 70%, respectively (Figure 1).

### Synteny between barley and rice – update

Barley-rice synteny was reassessed by comparing a high-density genetic map of barley comprising 1930 EST sequences with the annotated TIGR rice genome assembly 5. Barley-rice homologs were identified by sequence similarity comparison (BlastN, bit score ≥ 50) of the mapped barley EST sequences against all the rice CDS. In total, 1756 (91%) of the barley ESTs showed sequence homology with at least one rice CDS. EST sequences with homology to more than 5 non-locally duplicated rice genes were excluded from the analysis, leaving 1506 (78% of all) ESTs for further analysis. This step substantially reduced the 'background' that was not related to barley-rice synteny and was similar to the approach taken to filter background duplications within the rice genome. The genetic map position of each barley EST was plotted against the physical location of the highest BlastN-scoring rice homolog, which was considered the corresponding rice ortholog. Segments of the barley and rice genomes exhibiting conserved synteny were defined after visual inspection of the corresponding x-y plots allowing for small discrepancies of 5 cM from the colinear ordering of putative orthologs (Figure S3 of Additional file 1) to account for the limited resolution of the genetic barley consensus map (see Methods).

In total, 968 (50% of all) barley ESTs followed a colinear distribution with their corresponding rice homologs (putative orthologs). These were organized into 21 segments (Table S3 of Additional file 1), which we refer to as orthologous genomic regions. Shared synteny between both genomes spanned 993 cM (89%) of the barley genetic map and 236 Mb (63%, see Table S2 of Additional file 1) of the rice genome, respectively. No overlap between any two distinct regions of the barley genome to a single orthologous region in the rice genome and vice versa could be observed. Provided that our barley genetic map is sufficiently saturated with mapped ESTs, these findings imply that no additional subsequent large segmental duplication occurred after divergence of both species from their common ancestor.

Small genetic map intervals at proximal regions of barley chromosomes corresponded to relatively large physical intervals in rice and can be recognized by the sigmoidal shape in the x-y plots of orthologous gene positions in barley and rice (especially Hv3H and Hv6H, see Figure S3 of Additional file 1). For instance, a physical distance of almost 10 Mb of rice chromosome Os02 (position 8.5 Mb – 18.2 Mb) corresponded to a small interval of barley Hv6H at approximately 53 – 60 cM. On the extreme, both ends of rice chromosome Os01 shared synteny with two regions of barley chromosome Hv3H that were connected by cosegregating EST markers at ~69 cM. This low-recombining region that could not be resolved on the barley genetic map corresponded to the central 9.9 Mb – 22.3 Mb interval of rice Os01. Similarly, rice chromosomes Os08, Os10 and Os11 displayed synteny solely to the low-recombining regions of barley chromosomes Hv7H, Hv1H, and Hv4H (see Figure S3 of Additional file 1). This is in agreement with previously identified decreased recombination rates for proximal regions of barley chromosomes that often included centromeres [21].

**Figure 2:**
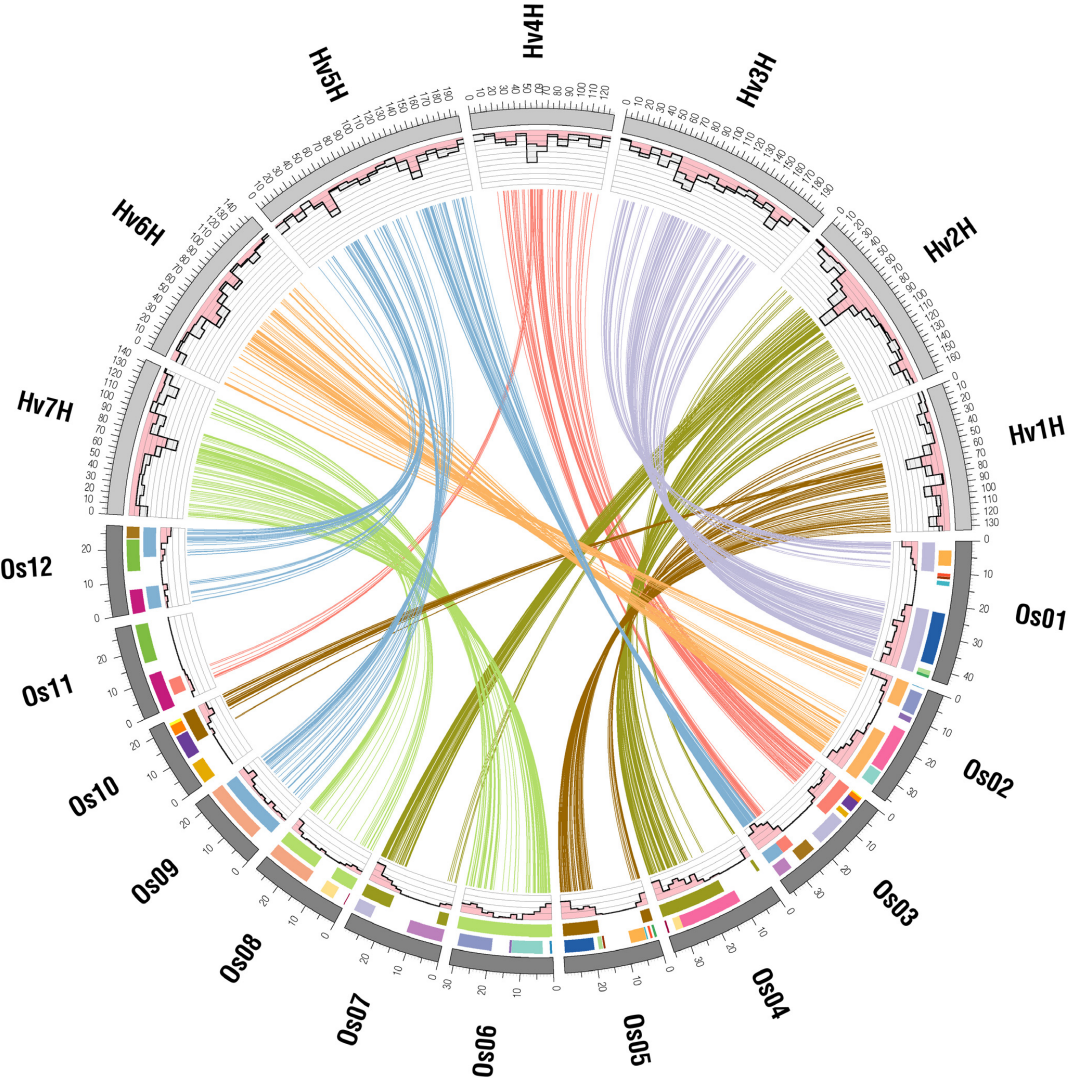
**Barley – rice synteny**. Best sequence similarities are displayed for syntenic barley ESTs placed on the seven barley chromosomes (Hv1H – Hv7H) to the rice genome (Os01 – Os12) by connecting lines. Histograms at barley chromosomes show the total number of markers and the proportion of those markers with synteny to rice in grey and red, respectively (10 cM intervals, scale: 0 – 50, scale spacing 5). Outer bars at the rice chromosomes show duplicated segment pairs within the rice genome (see Figure 1), inner bars indicate syntenic regions to the barley genetic map. The 12 histograms along the rice chromosomes show the ratio to the total number of annotated rice genes, which were syntenic to barley (scale: 0 – 0.08, scale spacing 0.02). Positions for rice are in Mb and for barley in cM.

The distribution of orthologs along the respective chromosomes of the barley genetic map and the rice genome sequence was biased. Rice orthologs of mapped barley ESTs were preferentially located at the ends of all rice chromosomes and contributed to more than 4% of all rice genes located in these regions (Figure 2, Figure S1 of Additional file 1). Establishing such a trend in the barley genome was difficult to achieve based on the availability of only a genetic map, because the exact physical dimensions of the low-recombining regions are difficult to determine and almost certainly explain the slightly higher ratio of syntenic barley ESTs versus all mapped ESTs in low-recombining regions (Figure 2).

### Shared patterns of whole-genome duplication between barley and rice

We observed widespread conservation of synteny between mapped barley ESTs and regions of the rice genome that were part of the rice WGD (Figure 2) providing indirect evidence for the WGD also having occurred in a common ancestor. The available integrated barley genetic map comprising 1930 EST sequences was not sufficiently dense to detect barley segmental genome duplications directly based on the sequence similarity of these mapped ESTs even at low BlastN stringency (bit scores ≥ 50). This was most likely due to the small number of paralogous gene pairs among the mapped barley ESTs and reflected in the fact that less than 10% of the rice genes effectively uncovered the rice WGD.

To obtain a genome-wide picture of the shared WGD pattern among barley and rice, we therefore took an indirect approach. We combined the fine-scale synteny data obtained for barley and rice and the bp-level coordinates of the rice WGD segments to precisely determine the shared ancestral duplications between barley and rice at a whole-genome level. Because of the chosen approach, shared ancestral duplicated blocks ("AD's") as observed in this study were restricted to the boundaries of duplicated segments in rice.

**Figure 3:**
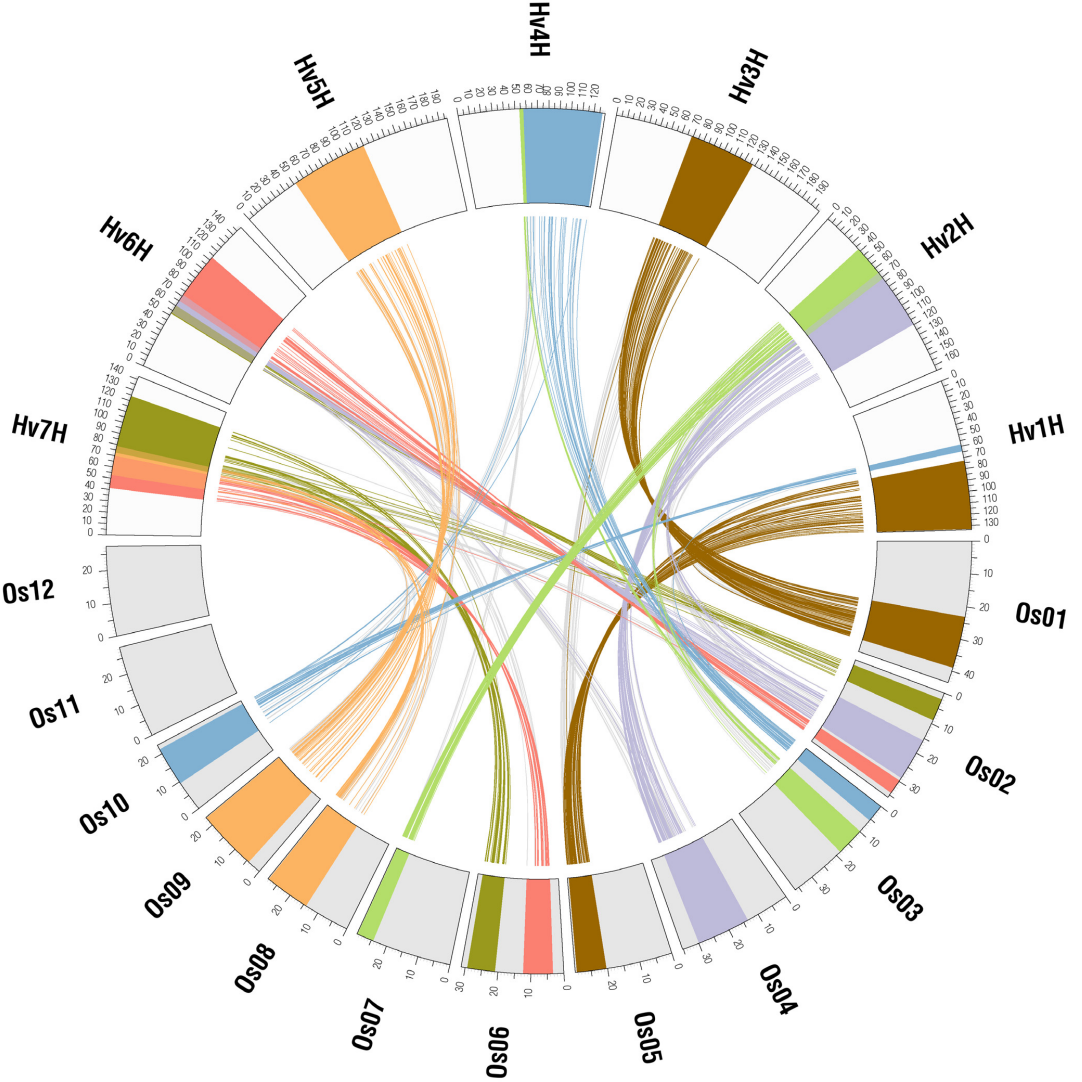
**Inferring barley genome duplications using the synteny to rice**. At least seven barley genome duplications could be inferred by analyzing the location of mapped ESTs among the seven barley chromosomes (Hv1H – Hv7H) and their best rice homologs (putative orthologs) among the 12 rice chromosomes (Os01 – Os12) with respect to rice segmental genome duplications (indicated by connecting lines of the same color). Relationships between second-best rice homologs (putative paralogs) of the same markers to the corresponding paralogous region in rice are shown in grey. Two hypothetical additional AD's involving Hv5H – Os03/Hv2H – Os07 and Hv4H – Os11/Hv5H – Os12 confirmed by less than 8 orthologous gene pairs to one of the duplicated rice regions are not shown.

**Figure 4:**
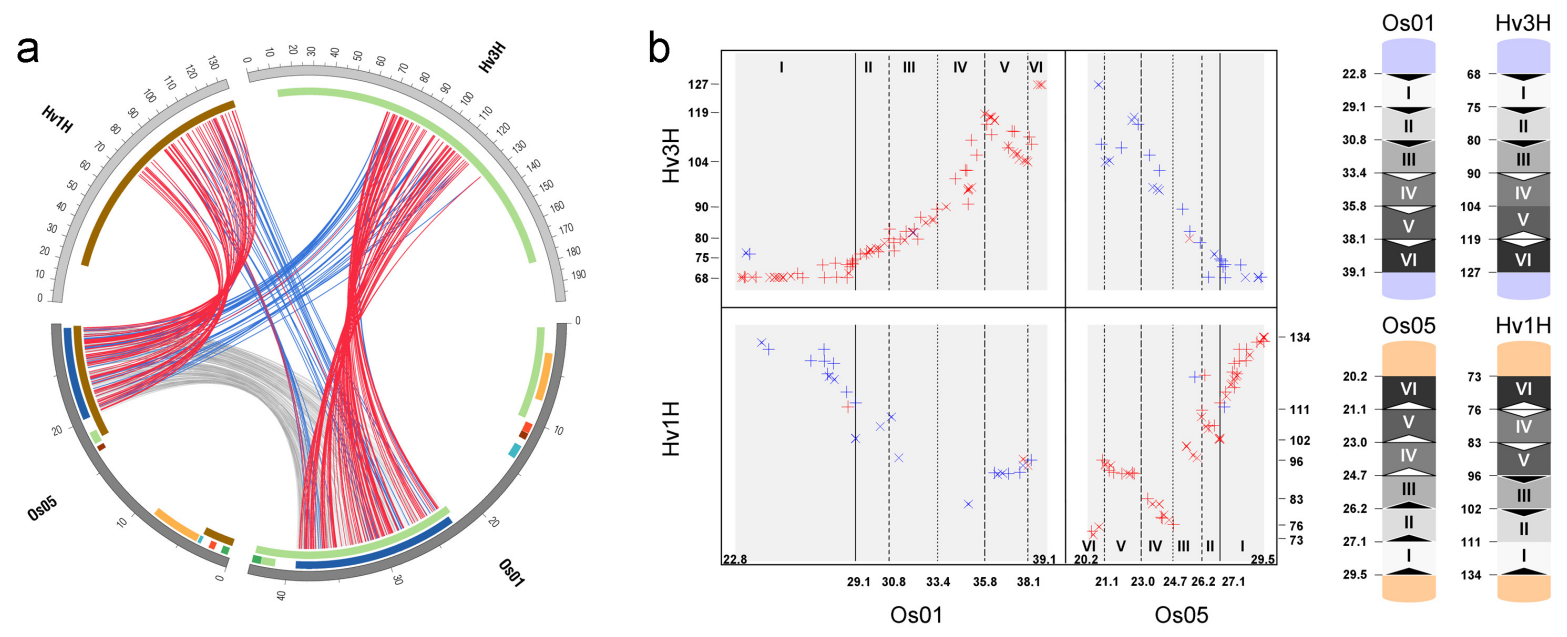
**Close up view on one ancestral duplicated block (AD_1) involving Hv1H/Hv3H and Os05/Os01**. a) A large degree of synteny existed between barley chromosome Hv1H and rice chromosome Os05 as well as between Hv3H and Os01 (indicated by brown and green inner bars). Grey-colored lines are shown between 354 segmentally duplicated rice genes located in the largest out of 7 duplicated segments between Os01 and Os05 (represented by colored bars in the outside lane). Best sequence homologies of barley ESTs to rice genes (putative orthologs) are represented by red and second best sequence homologies (putative paralogs) by blue connecting lines, respectively. b) Local chromosome inversions were identified in the corresponding orthologous regions of AD_1 by plotting the coordinates of mapped barley ESTs and the best (red) and second-best (blue) rice homologs against each other. Boarders of analogous subsegments I – VI corresponded to duplicated rice genes and are indicated by vertical lines. Although I – VI did not display inversions by themselves within the rice genome, inversions could be identified according to barley-rice synteny as represented schematically on the right panel of the figure. Positions are in Mb for rice and in cM for barley. Following gene pairs were used to define subsegments: LOC_Os01g50030 – LOC_Os05g47540, LOC_Os01g53079 – LOC_Os05g45320, LOC_Os01g57220 – LOC_Os05g42330, LOC_Os01g61420 – LOC_Os05g39380, LOC_Os01g65100 – LOC_Os05g35650.

Seven out of the 10 largest duplicated segments in rice shared synteny with barley and thus seven AD's could be identified (AD_1 to AD_7, Figure 3, Table S4 of Additional file 1). These corresponded to 584 (30%) of the 1930 mapped barley EST markers and covered 493 cM (44%) of the barley genetic map and 130 Mb (35%) of the rice genome. The number of identified orthologs corresponding to individual AD's ranged from 41 (AD_2) to 155 (AD_1). A bias in the number of identified orthologs among the two paralogous segments of a single AD was observed with ratios ranging from 1.4 (30/21) to 4.0 (48/12) for AD_5 and AD_7, respectively (Table S4 of Additional file 1). For AD_1 between rice chromosomes Os05 and Os01, the highest number of orthologous barley-rice gene pairs could be determined (Figure 4a). This included 63 barley EST sequences mapped to barley Hv1H (hv1H_os05_2) and 92 of Hv3H (hv3H_os01_2, see Table S3 of Additional file 1). This duplicated rice segment was the largest of five segmental duplications between Os01 and Os05 covering 354 duplicated gene pairs indicated by grey connecting lines (os01_05_5, Figure 4a, Table S1 of Additional file 1).

For more than 1/3 of all barley ESTs located in the seven AD's, 'second-best' rice homologs (putative paralogs) could be identified in addition to 'best' rice homologs (putative orthologs) (Figure 3). For almost all barley ESTs, both 'best' and 'second-best' rice homologs had been previously identified as paralogs in rice. For all AD's (AD_1 to AD_7), the distribution of 'best' and 'second-best' rice homologs of barley ESTs observed in the corresponding duplicated segments in rice was significantly different from random (one-sided Fisher's exact test, p < 0.05, Table S4 and Figure S4 of Additional file 1). Rare exceptions from this distribution were systematically analyzed for AD_1 (see Figure 4a). Here, four 'best' homologs (putative orthologs) were observed within the corresponding paralogous rice segments and five 'second-best' homologs (putative paralogs) within the corresponding orthologous rice segments (Table S4 of Additional file 1). Three possible reasons could explain these observations. First, for two barley EST-derived SNP markers (32_9757 and 32_9279), only a single homolog could be found in the rice genome and probably deletion of the corresponding orthologs after species divergence left their paralogs as 'best' homologs. Second, for RFLP marker GBR1769, assignment of the orthologous and paralogous rice homologs was supported by the significant difference in sequence homology (Blast e-value: 6.0E-177 versus 6.0E-149). Since this gene was mapped by RFLP hybridization [16], the mapped polymorphic DNA fragment of this multicopy probe most likely corresponded to a paralogous locus on Hv1H. Third, and in all other cases, the underlying barley EST was homologous to members of gene families, which were distributed across the rice genome.

Two hypothetical additional AD's were found for which less than 8 barley ESTs were syntenic to one of the duplicated segments in rice. In the first, shared synteny between Hv5H and Os03 was supported by 65 barley ESTs (hv5H_os03_1, Table S3 of Additional file 1) but only by 7 ESTs from Hv2H (hv2H_os07_2, Table S3 of Additional file 1) to the corresponding duplicated segment on rice Os07 (segment pair os03_07_2, R_1 in Figure S4 of Additional file 1). The second putative AD involved rice and barley chromosomes Os11 – Os12 and Hv4H – Hv5H. As the relationships between Hv4H – Os11 (hv4H_os11_1) and Hv5H – Os12 (hv5H_os12_1) were characterized by only 8 and 10 markers respectively, we consider that stronger support is required to strengthen the existence of this shared duplication.

### Analysis of synonymous substitution rates of rice genome duplications and barley-rice ancestral duplicated blocks

**Figure 5:**
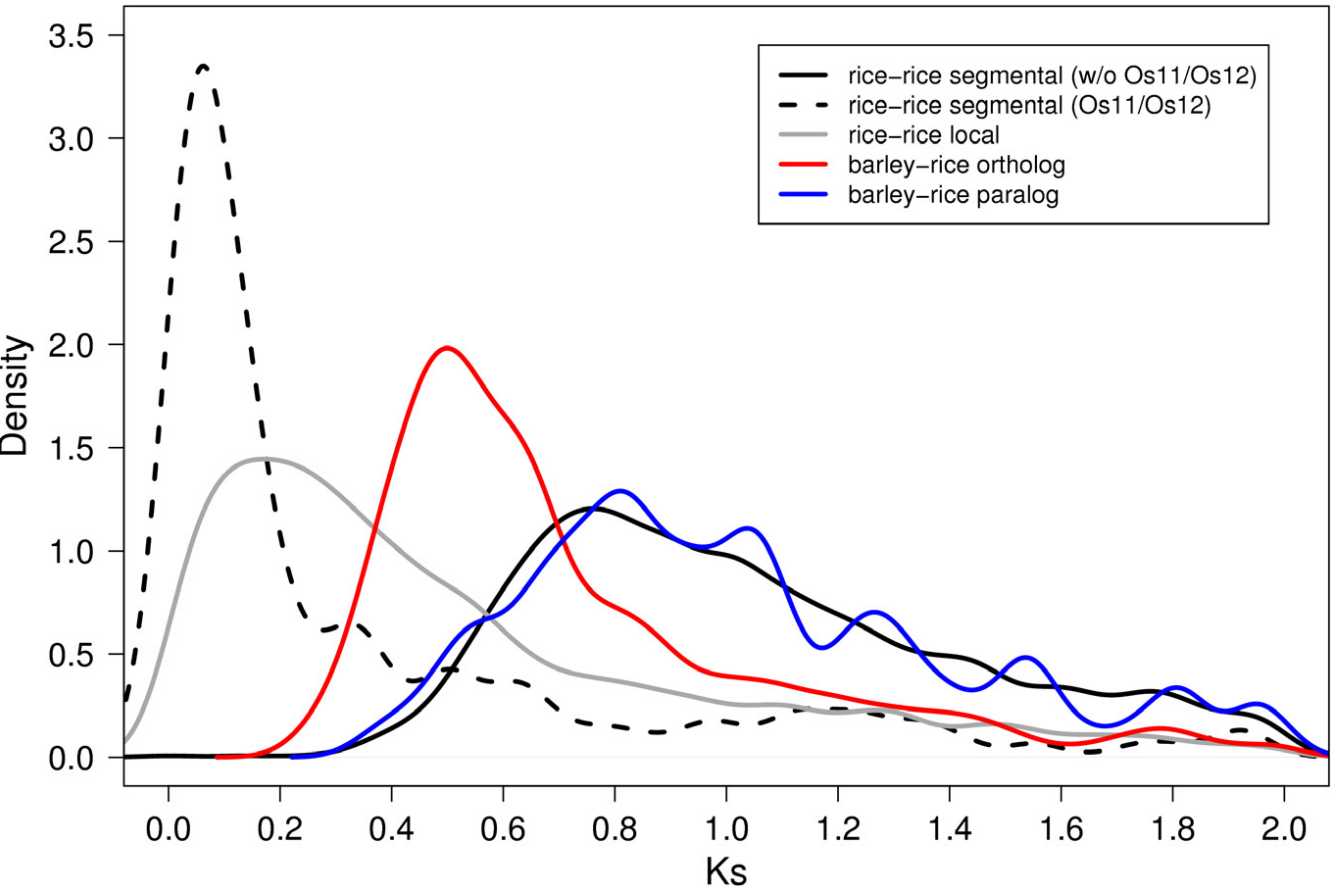
**Relative Ks dating of the WGD in rice and barley-rice species divergence**. Analysis of the Ks distribution among homologous gene pairs of the 10 largest duplicated segments (>10 Mb) in rice indicated the common origin of most segmental duplications (solid black color, based on Ks ratios of 1165 gene pairs) in a WGD and a more recent origin of the first duplicated segment between rice Os11 and Os12 (dashed black color, 244 gene pairs). The Ks distribution of barley-rice orthologous gene pairs (red, 816 gene pairs) suggested that WGD, although not directly detectable in barley, occurred in the common ancestor of both species, whereas gene homologs located at the ends of Os11 and Os12 together with local rice gene duplications (grey, 2433 gene pairs) were likely rice specific. The distribution of Ks ratios of paralogous barley-rice homologs (second-best non-locally duplicated rice homologs) among the identified seven ancestral duplicated segments is shown in blue (179 gene pairs).

We assessed, at genome scale, the time of origin of duplicated segments in rice for the ten largest segments (> 10 Mb) for which sufficient data was available to analyze the distribution of pairwise synonymous substitution rates (Ks) among corresponding duplicated genes. Duplicated genes of nine out of these ten segments had equivalent Ks distributions with similar median values ranging from 0.91 to 1.07 (Figure 1, Table S1 of Additional file 1). The means of those Ks distributions did not differ significantly (Tukey HSD test, p > 0.05) and resulted in a unimodal distribution with a single peak between 0.7 and 0.9 (median of 0.98) when plotted together in a single histogram (Figure 5).

Synonymous substitution rates of putative orthologs between barley and rice within AD's differed significantly from all distributions that were obtained for the different types of rice duplications (pair wise test on means, Tukey HSD, p < 0.05). A peak of the Ks distribution between 0.4 and 0.6 (median: 0.63, mean: 0.75) indicated that barley-rice orthologs were evolutionary younger than all segmental duplications in rice originating from WGD. However, they were older than the paralogs of the segmental duplication between chromosomes Os11 and Os12 (os11_12_1). The Ks distribution of putative paralogs between barley and rice with respect to ancestral duplications, was congruent (mean: 1.03, median: 0.95) with the distribution of synonymous substitution rates of the segmentally duplicated gene pairs (Figure 5, null hypothesis that means are different can not be rejected; Tukey HSD, p > 0.05).

### Traces of common and independent genome evolution within barley and rice

The distribution of segmental duplications within the rice genome uncovered a complex pattern of chromosomal rearrangements, which involved many if not all rice chromosomes as a consequence of diploidization after WGD in the ancestor genome. On the contrary, synteny to barley chromosomes could be detected for large segments or even complete rice chromosomes and was not restricted to boundaries of duplicated segments in rice. This observation suggested that major genome reorganizations took place after the WGD, but still before the barley-rice divergence. For instance, barley Hv6H shared synteny over the entire length of rice Os02, and only Os02, whereas rice chromosome Os02 consisted of segments that were duplicated on rice Os04 and Os06, respectively. Additional examples could be found for Hv3H/Os01 and Hv7H/Os06 (Figure 2).

**Figure 6:**
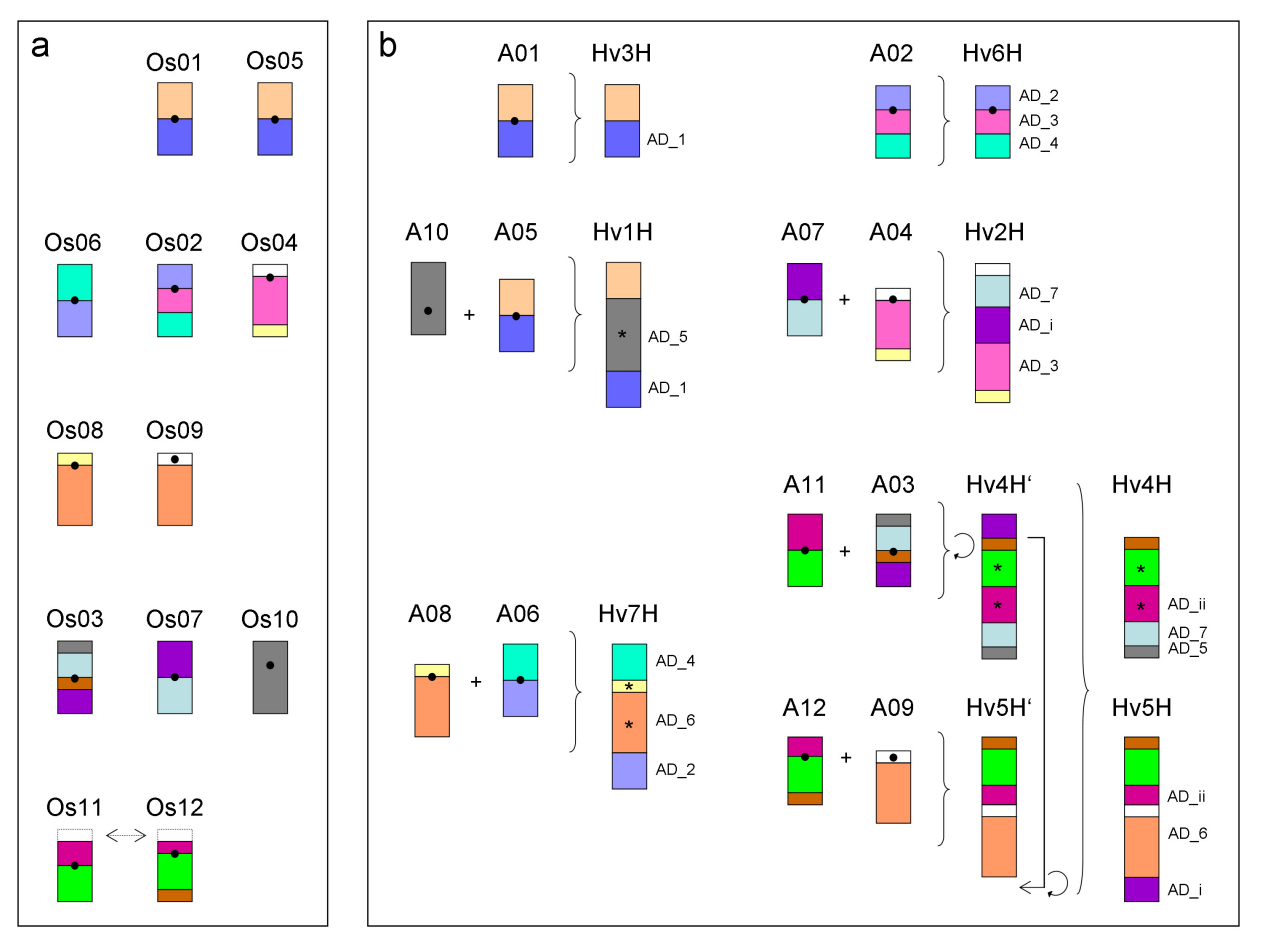
**Structural genome evolution model for barley from a rice-like ancestor with 12 chromosomes**. a) Schematic representation of 13 segmental duplications within the rice genome (involving together > 5 Mb). Each block among the rice chromosomes Os01 – O12 represents duplicated regions containing colinear gene pairs as highlighted by identical colors. Three adjacent, but inverted, segmental duplications between Os10 and Os03 are summarized in grey. Chromosomes were arranged according to their duplication pattern into five groups. Recent rice-specific gene conversion among the first few Mb of Os11 and Os12 are indicted by arrows [25]. b) Schematic representation of the structural model of the 7 barley chromosomes Hv1H – Hv7H that can be assembled by the repertoire of 12 rice-like ancestral chromosomes A01 – A12, which were named after the rice chromosomes. Barley Hv3H and Hv6H corresponded to the ancestral type of Os01 and Os02, respectively. The remaining barley chromosomes originated by combining two ancestral rice chromosomes (centromeres are indicated by black dots). Orthologous gene pairs provided evidence for the location of AD's (AD_1 to AD_7, see Figure 3 and Table S4 of Additional file 1). Two hypothetical additional AD's (AD_i and AD_ii) were confirmed by less than 8 orthologous gene pairs to one of the duplicated rice regions. Circular arrows indicate inversions. Asterisk symbols indicate cases, for which the orientation of ancestral chromosomes could not be identified in low-recombining central parts of barley chromosomes. After the divergence from rice, translocation of the distal segment corresponding to A03 from Hv4HS to Hv5HL took place in the Triticeae [15].

Based on the gross picture of synteny among barley and rice and keeping in mind the WGD pattern identified in the rice genome, a structural genome evolution model for barley was derived (Figure 6). It is based on (i) the above observation that major genome reorganizations took place before the divergence of the two species barley and rice and (ii) the reduction of chromosome numbers from 12 in rice to 7 in barley occurred by combining 10 ancestral rice chromosomes into 5 barley chromosomes. The two barley chromosomes Hv3H and Hv6H corresponded to the ancestral chromosome type of rice chromosomes (Os01 and Os02), subsequently described using the prefix 'A' (e.g. A01 is the ancestral type of rice chromosome Os01). The structure of the remaining barley chromosomes could be explained by combining the ancestral type of two rice chromosomes, whereas the first appears to be nested within the second: Hv1H = A10 → A05, Hv2H = A07 → A04, Hv4H = A11 → A03, Hv5H = A12 → A09, and Hv7H = A08 → A06 (Figure 6). After species divergence, barley along with other members of the Triticeae, underwent a lineage specific translocation of the distal part of the short arm of Hv4H to the long arm of Hv5H [15] (see Figure 6).

While long-range conservation of synteny among barley and rice was found to be well conserved, small inversions in one of the two species disturbed colinearity at Mb-scale. These included, for instance, the orthologous pairs hv2H_os04_1, hv4H_os03_1, and hv7H_os06_1 and involved not more than 5 Mb of the rice genome (Table S3 and Figure S3 of Additional file 1). AD_1 allowed a deeper analysis of small chromosomal rearrangements due to its higher marker coverage and good genetic resolution in the two corresponding barley segments (Figure 4b). Here, perfect colinearity was observed for the duplicated segments on Os01 (22.8 – 39.1 Mb) and Os05 (20.2 – 29.5 Mb) (os01_os05_5, Table S1 of Additional file 1). The order of corresponding barley-rice homologs, however, displayed a disruption of colinearity, which became visible after plotting the coordinates of homolog pairs against each other (red crosses in Figure 4b). A paracentric inversion could be observed for Hv3H (104 – 119 cM)/Os01 (35.8 – 38.1 Mb) and three paracentric inversions between Hv1H and Os05 (Figure 4b). For both orthologous segments, the pattern of synteny could be partitioned into six regions using the coordinates of duplicated rice genes located most closely to the break points of colinearity. By following this indirect approach, inversions could be related to paralogous segments between barley and rice and among the paralogous barley segments themselves (Figure 4b).

For rice genes located within duplicated genome segments, we observed a positive correlation of synteny to the barley genome and the retention of paralogous gene copies originating from WGD. Conservation of orthologous relationships can be related to paralog retention of the corresponding genes in barley. This was assessed in greater detail for AD_1, AD_3 and AD_6, which contained the highest numbers of barley-rice homologs (one-sided exact Fisher test, p < 0.05, Table S5 of Additional file 1). For instance, from the orthologous segment pairs on Hv3H and Os01 belonging to AD_1, 92 rice genes shared synteny with barley. Among these, 26 rice genes were duplicated on Os05, while the remaining 66 were not (ratio: 0.39). From the remaining 2409 rice genes embedded within this segment with no detectable synteny to barley, 328 were duplicated and 2081 were not duplicated in rice (ratio 0.16) and thus the odds ratio was significantly higher than 1 (one-sided Fisher test, p = 2.5E-04, see Table S5 of Additional file 1).

## Discussion

A WGD event in a common ancestor shaped the genomes of cereal crop species such as rice and Sorghum [10]. Footprints of this event have been detected previously by analyzing the complete genome sequence of rice [11,12]. By using colinearity between the rice genome sequence and gene-based genetic maps or genome-wide physical maps, traces of this ancestral WGD could also be found in maize [22], wheat [23,19] and barley [16]. In order to refine the picture of shared synteny between barley and rice, and relate this to the rice WGD footprint, we used an extensive gene-based genetic map of barley comprising ~2000 EST sequences.

### Footprints of WGD in rice: common consensus, but differences in detail?

We analyzed release 5 of the rice *japonica *genome obtained from TIGR and identified 24 distinct duplicated segments consisting of 4492 duplicated genes covering 261 Mb (70%) of the rice genome. The 1544 previously reported duplicated genes in ten duplicated blocks involving 45% of the rice genome [12] were consistent with our results, but the detection of almost three times as many colinearily-ordered duplicated genes in the present study allowed us to discover additional duplicated segments, for instance among Os03/Os12 and Os04/Os08 (Table S6 of Additional file 1). Of the 18 duplicated segments identified by Yu and co-workers [11], 17 corresponded to duplications identified in this study and explained 94% of the rice genome duplications that we observed. However, a direct comparison of the extent of duplicated segments would result in an underestimation of the true overlap mainly because of the different genome assemblies used in their study (*indica *versus *japonica*) that resulted in shifts of the coordinates of corresponding regions (77% overlap, Figure S5 and Table S6 of Additional file 1). Compared to their study, we did not find evidence for the duplication between Os04 and Os10, which appeared as the least supported duplication in terms of identified homologous pairs (5–15 depending on definition) and overlapped with larger (but not related) duplicated regions [11] (Figure S5 of Additional file 1). In addition to these previous studies, we identified several smaller duplicated segments that were located either at the ends of rice chromosomes (e.g. between Os04/Os08, Os02/Os06) or in close proximity to other duplicated regions suggesting pericentric inversions in one of the sister segments as an explanation for their location (e.g. between Os01 and Os05).

The ends of rice chromosomes Os11/Os12 differ from the other duplicated segments in the rice genome, both in terms of Ks distribution of gene-duplicate pairs and gene retention rate. It was previously suggested that this segmental duplication originated less than 5–8 million years ago [11,12,24] and emerged after the divergence of barley and rice. New findings suggested that both rice regions originated from the common cereal WGD, but were characterized by species-dependent concerted evolution acting independently in rice [25] and Sorghum [26]. Thus, the high sequence similarity between paralogs in these regions may reflect the age of gene conversion, rather than the age of segmental duplication. There are contrasting views about the existence of the corresponding regions in wheat [23,19]. We found evidence for the existence of both duplicated segments on barley chromosomes Hv4H and Hv5H supported by 8 and 10 syntenic homologs, respectively. This was in agreement with findings of one of the studies in wheat [23]. It is worth mentioning that orthologous segments were located in the rarely recombining central parts of the barley chromosomes (Figure 2, Figure 6) and not at the chromosome ends as in rice and Sorghum [26].

Local and background gene duplications are common in the rice genome [10,20]. Their identification and filtering are crucial steps prior to screening for gene duplications that originated from WGD. We utilized the genomic location and gene pair density of rice genes and their homologs to distinguish WGD-related segmental duplications from local and background duplications. Among the 56278 annotated rice genes of rice assembly 5 provided by TIGR, the Rice Genome Annotation group identified 15232 TE-related genes (including 15424 gene models). As non-directed gene duplication through transposon-like mechanisms could likely explain random background duplications identified by our approach, prior exclusion of identified TE-related genes could potentially simplify downstream analysis. However, only 17% (1986 out of 11686) of the genes involved in background duplications corresponded to TE-related genes. Therefore, pre-filtering of TE-related genes would not significantly reduce background duplications. Together with the observation that TE-related genes were significantly underrepresented in both segmentally (1.2%, 54 out of 4492) and locally duplicated genes (3.1%, 222 out of 7147) (one-sided Fisher's exact test: p < 2.2e-16), exclusion of rice TE-related genes would further have negligible impact on the identification of WGD-derived segments in rice and hence was not our method of choice. Previously described approaches to analyze the rice WGD included similar filtering steps, for instance using the location of duplicated genes in a visual approach using 2D scatter plots [11] or by focusing on gene duplications originating from similar time intervals estimated by analysis of Ks ratios [10]. Hence, there is consensus that sequence conservation among genes alone is not a reliable feature to unravel duplicated segments originating from WGD in rice. Moreover, this approach allowed us to identify 404 WGD-derived duplicated genes (10% of all 4492) that showed higher sequence similarity to more recent local duplications.

In a recent study by Salse and co-workers [19], rice genome duplications were analyzed using a previous rice annotation (TIGR release 4) and the program CloseUp [27]. Genome duplications were detected for as many as 20 newly defined chromosome pairs. These did, however, neither correspond to 3 additional chromosome pairings in a synteny study with maize [28] nor with previous literature [10-12]. Similarly, they were not supported by our study. In comparison to our results, differences in both the location and extent of duplicated segments in the rice genome were apparent, and these could not be simply explained by scaling or shifting of bp-coordinates, as for the previously mentioned *indica *assembly [11] (Figure S5 and Table S6 of Additional file 1). Further, some regions involved in the WGD appeared less extensive (e.g. Os02 – Os04, Os08 – Os09, Os11 – Os12), were not detected at all (e.g. Os04 – Os08, long arms of Os11 – Os12), or contradicted the WGD hypothesis because of overlaps between duplicated segments, e.g. for the long arm of rice chromosome Os02 (with duplicated segments on Os04 and Os06) or for two overlapping segments on Os06 duplicated on two distinct regions of rice Os02. Finally, we could not detect footprints of WGD in segments that contained the centromere. Neither did previous studies in rice [10] and *Arabidopsis *[2].

Arabidopsis underwent three successive rounds of WGD, which according to their time of origin and subsequent diploidization, represent different portions of the Arabidopsis transcriptome (83%, 51.6% and 20.3%) [29]. Ks analysis of the corresponding gene pairs supported this hypothesis [30]. In rice, contrasting views about the extent of genome duplications and their mode of origin have been discussed in the literature (for a review see [31]). One study [32] used a computational approach assuming a conserved order of paralogs to identify segmental duplications in the rice genome. They found a non-uniform distribution of segmental duplications across rice chromosomes involving not more than 20% of the genome. By additionally looking at Ks distribution of all duplicated genes [33], it was concluded that rice evolved from an ancient aneuploid rather than an ancient polyploid. Other studies [10-12] accumulated sufficient evidence for a WGD event explaining most of the genome duplications they observed in rice. In this study, we determined that more than 70% of the rice genome consisted of distinct duplicated segments characterized by colinearily-ordered duplicated gene pairs with a similar gene loss pattern and identical segment-wise Ks distributions. We therefore support the hypothesis of a single (detectable) ancient WGD, with rice (and barley) evolving from a tetraploid grass progenitor, which then diploidized by genome rearrangement and gene loss. We did not find evidence for colinear arrangement of higher-order homologs and thus did not find support for more than one ancient genome duplications in rice (data not shown). This result concurs with previous findings that failed to provide indications of multilevel duplication [11].

In contrast, Salse and co-workers [19] identified 16 duplicated regions superimposed upon duplicated regions involved in the WGD. Superimposition was prominent since 43%, 9% and 3% of the rice genome consisted of 2 times, 3 times and 4 times superimposed duplicated regions, respectively (computed from Supplemental Table 1 [19], see Table S6 of Additional file 1). Although those findings were evaluated as statistically significant by the CloseUp program [27], it remains debatable whether they reflect the true evolutionary history of the rice genome. The non-nested arrangement inside WGD-derived duplicated segments is incompatible with the hypothesis that the superimposed segments were remnants of more ancient segmental duplications as was suggested for Arabidopsis, where the β and γ segments were found to be nested inside the α segments [29]. If, however, such segments were of more recent origin, the number of duplicated genes located within them would be considerably higher. Also, it was reported that CloseUp assigned high statistical significance to cluster pairs that were probably spurious in a maize dataset and that the detection of homologous regions could have been obscured by random background matches [27]. Probably, very stringent sequence conservation parameters [19] alone were not sufficient to eliminate the influence of local and background duplications in rice and penalized less conserved gene pairs, as only 539 loci (as opposed to 2246 gene pairs in the present study) were found to be involved in segmental duplications, from which more than one third (201) contributed to gene pairs located on the first 6 Mb of Os11/Os12 (< 2% of the rice genome).

In summary, by analyzing the rice genome structure on an updated genome annotation, we found strong support for the commonly accepted single WGD hypothesis for the rice genome: (i) Segmental duplications involved more than 70% of the rice genome as described previously. (ii) No overlap among pairwise duplicated segments could be observed. (iii) Gene pairs located within corresponding sister segments were arranged in almost perfect colinear order. (iv) The duplicated genes of nine out of the ten largest duplicated segments comprised equivalent Ks distributions and gene retention rates suggesting a common origin.

### Shared patterns of WGD between barley and rice suggest a structural evolution model of the barley genome

The existence of shared patterns of a common WGD predating the divergence of barley and rice was supported by three observations in our data: (i) The pattern of macro-synteny between barley and rice was highly conserved. This implied that genome reorganization after WGD occurred in the common ancestor of both barley and rice before species divergence. (ii) Synteny between barley and rice involved the largest duplicated rice regions and for at least seven of these paralogous relationship in barley could be resolved to segments that were orthologous to duplicated regions in the ancestral grass genome progenitor. (iii) Smaller Ks of barley-rice orthologs compared to paralogs located in WGD-derived segments in rice confirmed a WGD before species divergence.

The roughly 2000 gene-based genetic map of barley provided sufficient coverage to explain genome wide shared synteny between barley and rice. The use of classical genetic maps in the presented study was however limited in the regions that correspond to central/centromeric parts of barley chromosomes. These regions are characterized by low recombination rates over large physical distances. Resolution in these regions is poor and while the corresponding syntenic regions could be identified to the rice genome (e.g. for Os11/Os12), inversions or even the orientation of whole syntenic rice chromosomes could not be fully resolved.

We found excellent correspondence to the grass genome colinearity model proposed by Devos [15]. Using colinearity to the rice genome, three recent studies suggested partly different grass genome evolution models for wheat [22,23,19], which shares a close evolutionary relationship to barley. We found concordance with the first study [22] except for the long arm of chromosome 5, for which synteny was detected to Os10 and not to Os03 as in our study. Differences to the second study [23] regard chromosome 2 for which we detected synteny to Os04. In contrast to the third wheat study [19], our data suggested correspondence of the Triticeae specific translocated segment between barley Hv4H and Hv5H [15] to the duplication between rice Os03 and Os07 (os03_07_2, Table S1 of Additional file 1) and not to the duplicated segments between Os11 and Os12 for which we identified synteny to central parts of barley Hv4H and Hv5H (hv4H_os11_1 and hv5H_os12_1, Table S3 of Additional file 1) at orthologous positions. Further differences exist in the detection of duplications between barley Hv6H and Hv7H (AD_2 and AD_4, see Table S4 of Additional file 1 and Figure 6) that were not detected between the corresponding wheat chromosomes [19].

The mosaic of syntenic segments between barley and rice in the present study resulted in a karyotype evolution model for the structurally similar Triticeae genomes (Figure 6) in which five barley chromosomes represent combinations of two ancestral chromosomes. Our data suggest a characteristic pattern of the respective 'fusion' chromosomes in which one ancestral chromosome was enclosed by a second ancestral chromosome evolving probably by similar mechanisms. Similarly, reduction of chromosome numbers in Sorghum (10) compared to rice (12) could be explained by combining Os09 and Os07 in Sorghum chromosome SB and Os10 and Os03 in SC [10]. Pericentric inversions and translocations between two ancestral chromosomes with terminal and pericentric breakpoints and subsequent loss of a minichromosome as one of the translocation products could most parsimoniously explain chromosome reduction in barley, similar as suggested for chromosome reduction within the Brassicaceae family ([34], for review see [35]). Together with inversions and the previously described translocation of the end of the short arm of Hv4H to the long arm of Hv5H within the Triticeae [15], the genome structure of barley could be explained by a collection of simple evolutionary mechanisms that retain a high degree of structural conservation with rice after the taxon divergence.

## Conclusion

A barley genetic map consisting of approximately 2000 non-redundant EST sequences allowed us a detailed analysis of shared synteny with the rice genome. Based on shared synteny between both species, the locations of at least seven corresponding duplicated segments were identified that originated from an ancestral WGD event. While small paracentric inversions and reduction of chromosome numbers were species specific, genome reorganization after WGD preceded taxon divergence as shared synteny extended the boundaries of rice duplicated genomic segments originating from WGD and only low levels of differential gene loss were found. Our results indicate positions of WGD segments within the barley genome that are shared with rice and hence the potential location of putative barley paralogs. Comparative analysis with the rice genome further showed that low-recombining central parts of barley chromosomes, although not represented by many genetic markers, can correspond to large physical regions of the rice genome consisting of many genes.

## Methods

### Rice data set

Release 5 of the Rice Pseudomolecules and Genome Annotation [36] was used and downloaded from the Rice Genome Annotation Project [37]. In total, annotated coding sequences (CDS) of 66710 gene models from 56278 predicted genes were readily usable in FASTA format (all.cds) for further analysis. Locus names of TE-related gene models were extracted from the file all.TE-related. Chromosome locations as well as start and end coordinate of the respective gene models were extracted from the file all.TU_model.brief_info.5.0 covering 372 Mb of the rice genome. The center of the start and end coordinate was computed in order to represent gene positions by a single value.

### Barley data set

Two sources of barley genetic markers were utilized and integrated. The first set, subsequently referred to as 'map1', originated from a previously published barley consensus transcript map, which combined genetic positions of cDNAs from three individual mapping populations using RFLP, SNP, and SSR marker technologies [16]. Redundancy in the data was eliminated for 11 duplicates and 2 triplicates whenever different markers related to the same cDNA lead to identical genetic positions (allowing an error of maximal 5 cM for the consensus map). Genetic mapping of 1024 cDNA clones resulted in a total of 1047 genetically mapped loci. In 13 cases cDNA clones were mapped to 2 different loci and in 5 cases to 3 different loci comprising a minimum distance to each other of more than 5 cM when placed onto identical chromosomes. At least one representative EST sequence was available for each mapped cDNA clone, which could be used to identify homologs in the rice genome. However, for 788 cDNA clones both, 5'- and 3'-ESTs, were available and were used together for further analysis summing up the total number of ESTs derived from all mapped cDNA clones to 1812.

A second unpublished set, subsequently referred to as 'map2', comprised 1025 EST-based SNP loci derived from a doubled haploid (DH) 'Morex' × 'Barke' population (211 individuals corresponding to a resolution of ~0.5 cM) mapped by the Illumina Golden Gate assay (Illumina Inc., San Diego, USA) as described previously [38] (data can be obtained from the authors). Marker identifiers were linked to barley EST consensus sequences of the HarvEST assembly #32 from 03/03/03 comprising 351645 public ESTs [39].

In order to integrate both marker datasets ('map1' and 'map2') into a more comprehensive consensus map, gene sequence information underlying all markers was referenced against a single tentative barley unigene set based on assembly #32 of the HarvEST database [39]. As a result, 158 markers of map1 matched the same HarvEST unigene as 142 markers of map2. In some cases, two or more ESTs that mapped to different locations in the barley genome corresponded to a single HarvEST unigene. These were excluded from a final set of 131 common markers that were used as anchor points for map integration. These anchors varied in number from 13 on chromosomes Hv1H and Hv4H to 29 on chromosome Hv2H (Table S7 of Additional file 1).

Three regression methods implemented in the R statistical language [40] were tested for their applicability to normalize observed map length differences of up to 24% among chromosomes of both datasets using the previously defined anchor points (Figure S6 of Additional file 1):

(i) Linear least squares regression implemented in the R function *lm*

(ii) Local polynomial regression fitting using *loess *with the *degree = 1 *(the degree of polynomials) and *span = 0.7 *(the smoothing parameter) after inspection of the resulting plots with respect to the accurate placing of telomeric and pericentromeric markers

(iii) Local polynomial regression fitting with the same set of parameters as (ii), but as an robust implementation using the Huber weight function, which depends on the residuals of the anchor points and therefore is less sensitive to outliers. In the initial state, for each anchor marker a weight of 1 was taken. In an iterative approach weights were recalculated depending on the coefficients of the regression and the resulting residuals according to the Huber weight function , whereas *e *is the residual and *k *the tuning constant, which is computed with *k *= 1.345 *MAR*/0.6745 and *MAR *is the median absolute residual [41]. Ten iterations were sufficient to obtain a stable estimate of the regression coefficients.

The robust implementation of the local polynomial regression fitting (*loess*) using the Huber weight function outperformed the non-robust implementation of *loess *as well as the linear regression fitting in terms of robustness against outliers and mean prediction error (Table S7 of Additional file 1). As a result, the remaining 883 markers of dataset 2 could be integrated into dataset 1 resulting in an integrated transcript map consisting of 1930 genes covering 1118 cM.

### Identification of homologs

In order to identify homologous genes based on sequence similarities both among all rice CDS themselves (rice-rice) as well as among barley cDNA sequences and rice CDS (barley-rice), a BlastN analysis [42] was performed using version 2.2.13 provided by NCBI using an E-value significance threshold of 1e-5 (rice-rice) and 1e+1 (barley-rice), respectively. Several post-processing steps were done with the help of a Perl program. Blast hits not exceeding a Blast bit score of 100 (rice-rice) or 50 (barley-rice) were excluded as well as hits to the same gene (rice-rice). With the aim to identify rice-rice homologs (including all CDS of gene models of a single gene) or barley-rice homologs (including all available EST sequences corresponding single barley marker and involving all CDS of its gene models), all Blast hits were pooled and the highest scoring Blast alignment was chosen, respectively. Such homologs were grouped into clusters of locally duplicated genes whenever they were located adjacently (≤ 1 Mb) to another member of the same cluster. Among such clusters, the homolog showing the highest scoring alignment was labeled *non-locally duplicated *towards the gene of interest. To account for the possibility that genes were duplicated in a WGD and subsequently could be involved in a recent local duplication thus showing higher sequence similarity to the gene of interest, we selected best sequence similarities to a single member of a *non-locally *duplicated gene cluster. However, in the rice-rice analysis, all members of such a homolog cluster were labeled *locally duplicated *if the gene of interest was located within 1 Mb of any of the homologs of that cluster. In order to exclude higher-order homologs from the analysis, all genes comprising more than 5 non-locally duplicated homologs were discarded.

To reduce the background noise that we observed for homolog pairs in the rice genome, a density based approach was chosen to compute scores for each homologous pair that could be placed according to their coordinates in a 2D-matrix in the following way:

(1) For each given homolog pair *x*, identify all additional homolog pairs *N *in the neighborhood that are located within a Euclidian distance *d*(*x*, *n*) of k = 0.05 of the respective chromosome sizes

(2) Compute a distance-dependent score for *x *using the location of all neighbors as: 

(3) Discard all *x *with *W *< 2 as background noise

Subsequently, the remaining rice gene pairs were grouped into segments fulfilling the following requirements: The distance between direct neighbors of homologous pairs is ≤ 500 kb, the minimum number of homologous pairs per segment is 5, and the minimum size of the segment is at least 500 kb on both chromosomes.

All circular figures were generated with the help of the software Circos v 0.22 [43].

### Statistical tests

The one-sided Fisher's exact test implemented in the function *fisher.test *of the R statistical package was applied to test (i) the distribution of homologs located in duplicated segments in the rice genome under the null hypothesis H_0 _that there is no overrepresentation of homologs within both rice intervals, (ii) the distribution of orthologs between barley and rice under H_0 _that there is no overrepresentation of 'best' homologs within both barley and rice intervals and (iii) the distribution of 'best' and 'second-best' rice homologs of given barley ESTs across ancestral duplicated blocks under H_0 _that there is no overrepresentation of 'best' homologs in the putative orthologous and no overrepresentation of 'second-best' homologs in the putative paralogous regions. In all cases H_0 _was rejected for p ≤ 0.05.

The Tukey Honest Significant Difference method implemented in the function *TukeyHSD *of the R statistical package was used to compute confidence intervals of pairwise means among the different data sets to test which means of the Ks distributions differ significantly.

### Ks dating

In order to obtain a frameshift-unbiased codon alignment and hence a more reliable estimate about the distribution of Ks values between mapped barley ESTs and rice CDS, a pipeline was implemented in the programming language Perl utilizing the two external programs *bl2seq*, which allows Blast on 2 sequences ([42], NCBI Blast version 2.2.13), and *yn00 *from the PAML package ([44,45], version 3.15 from November 2005). The Blast parser used for accessing the Blast outputs as well as the PAML wrapper and parser necessary for obtaining Ks values according to Yang and Nielsen [46] was done with the help of several BioPerl packages ([47], version 1.5.2. from December 2006). Input files were a single file containing the identifiers of homologs to be analyzed and two FASTA files, one containing the barley EST sequences with an assumed low sequence quality introducing frameshifts and the other containing the annotated rice CDS starting in-frame with the first codon of the predicted rice gene.

The prerequisite step to obtain estimates on Ks values of any given homologous pair of sequences is a reliable codon alignment. An initial codon-usage unbiased alignment was obtained by extracting the highest-scoring alignment (of possibly multiple alignments) of a TBlastX analysis. However, TBlastX is unable to perform gapped alignments and thus frameshifts caused by sequencing errors of 1 or 2 bases within the EST sequence could lead to a suboptimal or shortened alignment. For this reason, a subsequent gapped BlastN analysis of the same sequences was performed and the resulting alignment was scanned for gaps. If gaps were found, the original sequences were modified depending on which sequence strand such gap characters could be found within the alignment: On the one hand, all nucleotides of the EST sequence located opposite to connected gaps in the CDS are either frameshifts (if the number is not a multiple of 3) or inserted codons (which are not relevant for the calculation of Ks) and hence were cut out. On the other hand, gaps within the EST alignment string were correlated to the respective codon in the CDS (all CDS are in-frame) and all such codons in the CDS as well as all corresponding nucleotides of the EST were cut out and thus no frameshift was introduced into the CDS.

A second TBlastX alignment was performed to test whether these modifications lead to a better codon alignment in terms of E-value and Bitscore. Then, the codon alignment was checked for two essential quality criteria, for the absence of internal STOP codons within the EST sequence and for the +1 frame of the CDS sequence. The last codon pair of the alignment was chopped in case of a terminal STOP codon within the CDS sequence. After all quality checks were fulfilled, the resulting codon alignment was handed to the software *yn00 *for obtaining the Ks substitution ratio utilizing BioPerl objects and modules.

Computation of Ks substitution rates of the 'map1' marker data set were based on respective HarvEST consensus sequences extracted according to EST cluster membership. Ks values were computed for all different data sets and Ks values > 2 were not retained. Density plots of Ks distributions were plotted by applying a smoothing bandwidth of 0.05.

## List of abbreviations

AD: ancestral duplicated block; CDS: coding sequence; EST: expressed sequence tags; Hv: *Hordeum vulgare*; Ks: synonymous substitution rates; Os: *Oryza sativa*; RFLP: restriction fragment length polymorphism; SNP: single nucleotide polymorphism; TE-related genes: transposable element-related genes; Tukey HSD: Tukey's Honest Significant Differences test; WGD: whole-genome duplication.

## Authors' contributions

TT, AG, IG and NS conceived the study. TT designed the study, analyzed the data and wrote the manuscript. NS computed the genetic map on unpublished barley genotyping data provided in collaboration with RW and TJC. AG, RW and NS contributed to the writing of the manuscript. All authors read and approved the final paper.

## Supplementary Material

Additional file 1 **Supplementary Figures and Tables**. This file is a collection of supplementary Figures and Tables. Click here for file
