# Supplementary material for: Evidence and evolutionary analysis of ancient whole-genome duplication in barley predating the divergence from rice
Source: BMC Evol Biol. 2009 Aug 22;9:209. doi: 10.1186/1471-2148-9-209 (PMC2746218; doi:10.1186/1471-2148-9-209)
Supplement: Additional file 1 — Supplementary Figures and Tables. This file is a collection of supplementary Figures and Tables. [file 1471-2148-9-209-S1.pdf]

Evidence and evolutionary analysis of ancient whole-genome duplication in barley predating the divergence from rice

Thomas Thiel, Andreas Graner, Robbie Waugh, Ivo Grosse, Timothy J Close, Nils Stein

Additional file 1

**Figure**	**S1:**	**Combined density plots for the distribution of subsets of rice genes**	**2-4**
**Table**	**S1:**	**Duplicated segments within the rice genome**	**5**
**Figure**	**S2:**	**Duplicated segments within the rice genome**	**6-17**
**Table**	**S2:**	**Extent of rice segmental duplications and shared synteny with barley**	**18**
**Figure**	**S3:**	**Shared synteny between barley and rice**	**19**
**Table**	**S3:**	**Shared synteny between barley and rice**	**20**
**Table**	**S4:**	**Shared ancestral duplications between barley and rice**	**21**
**Figure**	**S4:**	**Shared ancestral duplications between barley and rice**	**22**
**Table**	**S5:**	**Positive correlation of shared synteny with barley and retention of WGD paralogs in rice**	**23**
**Table**	**S6:**	**Comparison of location and extent of rice segmental duplications to previous studies**	**24**
**Figure**	**S5:**	**Comparison of location and extent of segmental duplications within the rice genome to two previous studies**	**25**
**Table**	**S7:**	**Mean prediction errors of regression methods used for barley map integration**	**26**
**Figure**	**S6:**	**Regression plots for the integration of two barley genetic maps**	**27-33**

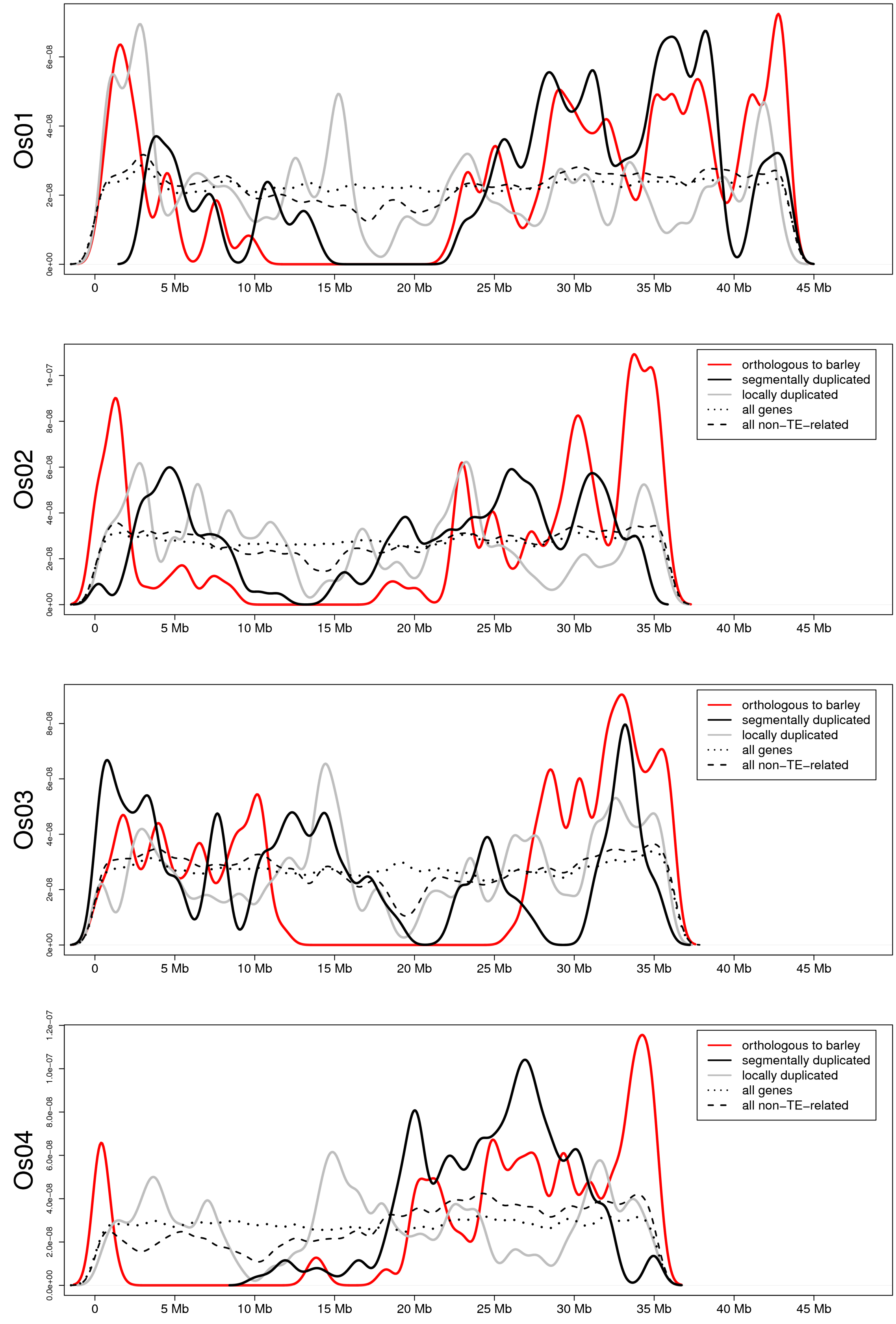

Figure S1: Combined density plots for the distribution of subsets of rice genes. Density curves were computed along rice chromosomes Os01 – Os12 using a smoothing bandwidth of 0.5 Mb for the gene subsets (1) shared synteny with barley, (2) segmentally duplicated, (3) locally duplicated within the rice genome in comparison to all genes and all non-TE-related genes.

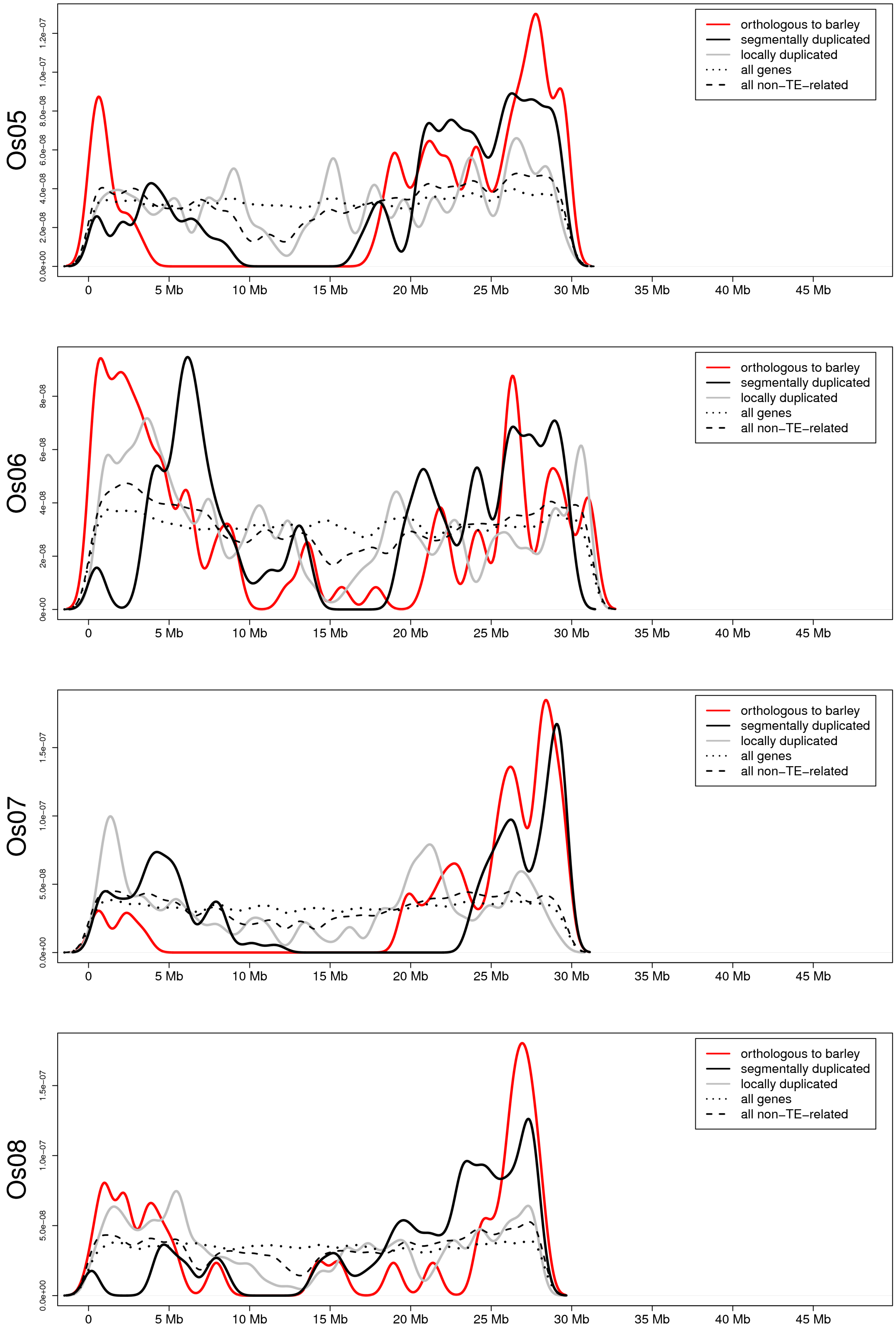

Figure S1 (continued)

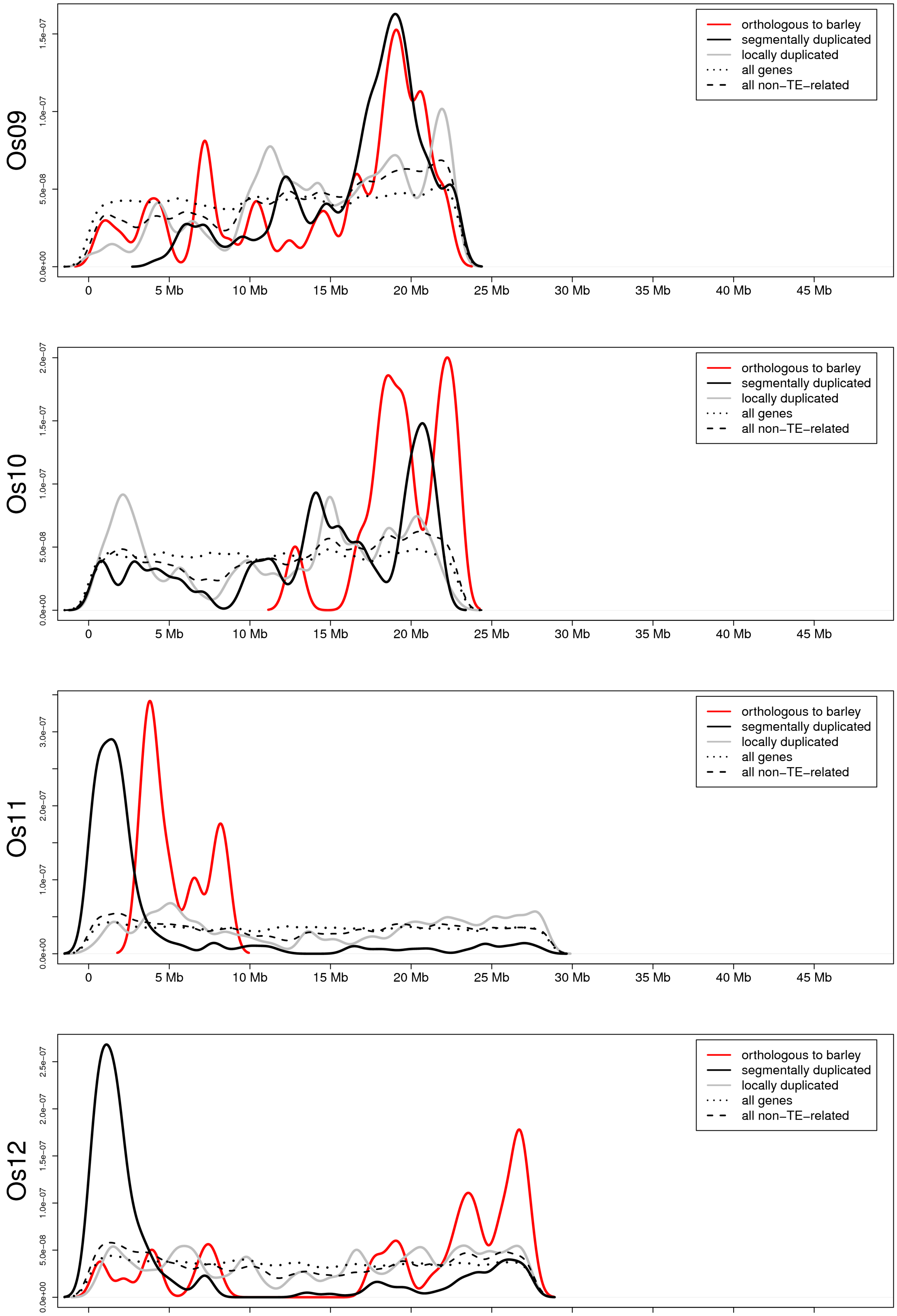

Figure S1 (continued)

Table S1: Duplicated segments within the rice genome.

	segment 1								segment 2								nr		
	chromo-	start		end		size	nr of	ratio	chromo-	start		end		size	nr of	ratio	duplicated		Ks
name	some	(Mb)	TIGR locus ID	(Mb)	TIGR locus ID	(Mb)	genes	duplicates	some	(Mb)	TIGR locus ID	(Mb)	TIGR locus ID	(Mb)	genes	duplicates	genes	significance [1]	median [2]
os01_05_1	Os01	3.0	Os01g06270	7.7	Os01g13770	4.7	704	10%	Os05	3.6	Os05g06980	8.6	Os05g15160	4.9	712	9%	67	< 2.2E-16	
os01_05_2	Os01	10.2	Os01g18240	11.2	Os01g19694	1.0	136	10%	Os05	2.3	Os05g04820	1.7	Os05g03884	0.6	91	15%	14	< 2.2E-16	
os01_05_3	Os01	11.2	Os01g19760	11.9	Os01g21320	0.7	103	8%	Os05	16.8	Os05g28710	17.3	Os05g29990	0.5	79	10%	8	< 2.2E-16	
os01_05_4	Os01	12.6	Os01g22520	14.0	Os01g24880	1.4	218	6%	Os05	3.5	Os05g06750	3.2	Os05g06270	0.3	49	27%	13	< 2.2E-16	
os01_05_5	Os01	22.8	Os01g39780	39.1	Os01g66820	16.4	2501	14%	Os05	29.5	Os05g51540	20.2	Os05g34270	9.3	1570	23%	354	< 2.2E-16	0.95
os01_05_6	Os01	41.2	Os01g70580	42.6	Os01g72790	1.4	210	12%	Os05	17.6	Os05g30454	18.8	Os05g32360	1.2	184	14%	25	< 2.2E-16	
os01_05_7	Os01	42.6	Os01g72890	43.5	Os01g74510	0.9	161	11%	Os05	0.3	Os05g01540	1.0	Os05g02790	0.7	124	14%	17	< 2.2E-16	
os02_04_1	Os02	14.7	Os02g25230	28.9	Os02g47410	14.3	2097	14%	Os04	10.6	Os04g19140	30.2	Os04g51370	19.6	2935	10%	288	< 2.2E-16	1.01
os02_06_1	Os02	0.1	Os02g01270	0.3	Os02g01520	0.2	27	26%	Os06	0.9	Os06g02590	0.3	Os06g01500	0.6	103	7%	7	< 2.2E-16	
os02_06_2	Os02	1.9	Os02g04230	9.0	Os02g15880	7.1	1053	17%	Os06	30.0	Os06g49430	19.3	Os06g33180	10.6	1585	11%	177	< 2.2E-16	0.91
os02_06_3	Os02	10.0	Os02g17350	11.7	Os02g19860	1.7	244	3%	Os06	13.2	Os06g22650	13.7	Os06g23530	0.6	81	10%	8	< 2.2E-16	
os02_06_4	Os02	29.5	Os02g48190	34.3	Os02g56140	4.9	788	17%	Os06	13.0	Os06g22330	3.3	Os06g06900	9.7	1447	9%	132	< 2.2E-16	1.01
os03_07_1	Os03	9.7	Os03g17570	19.2	Os03g33590	9.4	1399	10%	Os07	29.6	Os07g49460	23.9	Os07g39900	5.7	921	16%	146	< 2.2E-16	0.98
os03_07_2	Os03	31.1	Os03g54850	35.7	Os03g63330	4.6	805	13%	Os07	0.5	Os07g01850	11.9	Os07g20544	11.4	1737	6%	102	< 2.2E-16	0.99
os03_10_1	Os03	0.1	Os03g01150	0.5	Os03g01820	0.4	69	25%	Os10	21.9	Os10g41340	21.0	Os10g39810	0.9	145	12%	17	< 2.2E-16	
os03_10_2	Os03	0.5	Os03g01880	2.1	Os03g04480	1.5	242	19%	Os10	17.7	Os10g33900	21.0	Os10g39790	3.3	526	9%	46	< 2.2E-16	
os03_10_3	Os03	2.1	Os03g04500	5.7	Os03g11100	3.6	590	12%	Os10	17.2	Os10g33290	9.4	Os10g19190	7.7	1150	6%	68	< 2.2E-16	0.98
os03_10_4	Os03	6.6	Os03g12550	8.2	Os03g15050	1.6	236	15%	Os10	0.4	Os10g01610	7.2	Os10g13700	6.8	1026	3%	35	< 2.2E-16	
os03_12_1	Os03	22.0	Os03g39680	27.6	Os03g48560	5.6	796	8%	Os12	23.8	Os12g38740	27.4	Os12g44240	3.6	540	11%	61	< 2.2E-16	
os08_04_1	Os08	0.1	Os08g01160	0.3	Os08g01410	0.2	27	22%	Os04	34.7	Os04g58760	35.2	Os04g59550	0.5	75	8%	6	< 2.2E-16	
os08_04_2	Os08	4.1	Os08g07400	8.6	Os08g14330	4.4	671	5%	Os04	32.5	Os04g55030	30.2	Os04g51390	2.3	365	10%	35	< 2.2E-16	
os08_09_1	Os08	13.8	Os08g23150	28.1	Os08g45010	14.3	2120	11%	Os09	4.2	Os09g08130	22.9	Os09g39910	18.7	2814	8%	228	< 2.2E-16	0.97
os11_12_1	Os11	0.0	Os11g01020	12.0	Os11g20790	12.0	1817	19%	Os12	0.0	Os12g01010	7.5	Os12g13380	7.5	1188	30%	352	< 2.2E-16	0.13
os11_12_2	Os11	15.9	Os11g28520	28.1	Os11g47460	12.2	1742	2%	Os12	13.3	Os12g23490	23.3	Os12g37970	10.0	1379	3%	40	< 2.2E-16	1.07

[1] one-sided Fisher test, see Methods for details

[2] median Ks ratios for the ten largest duplicated blocks in the rice genome

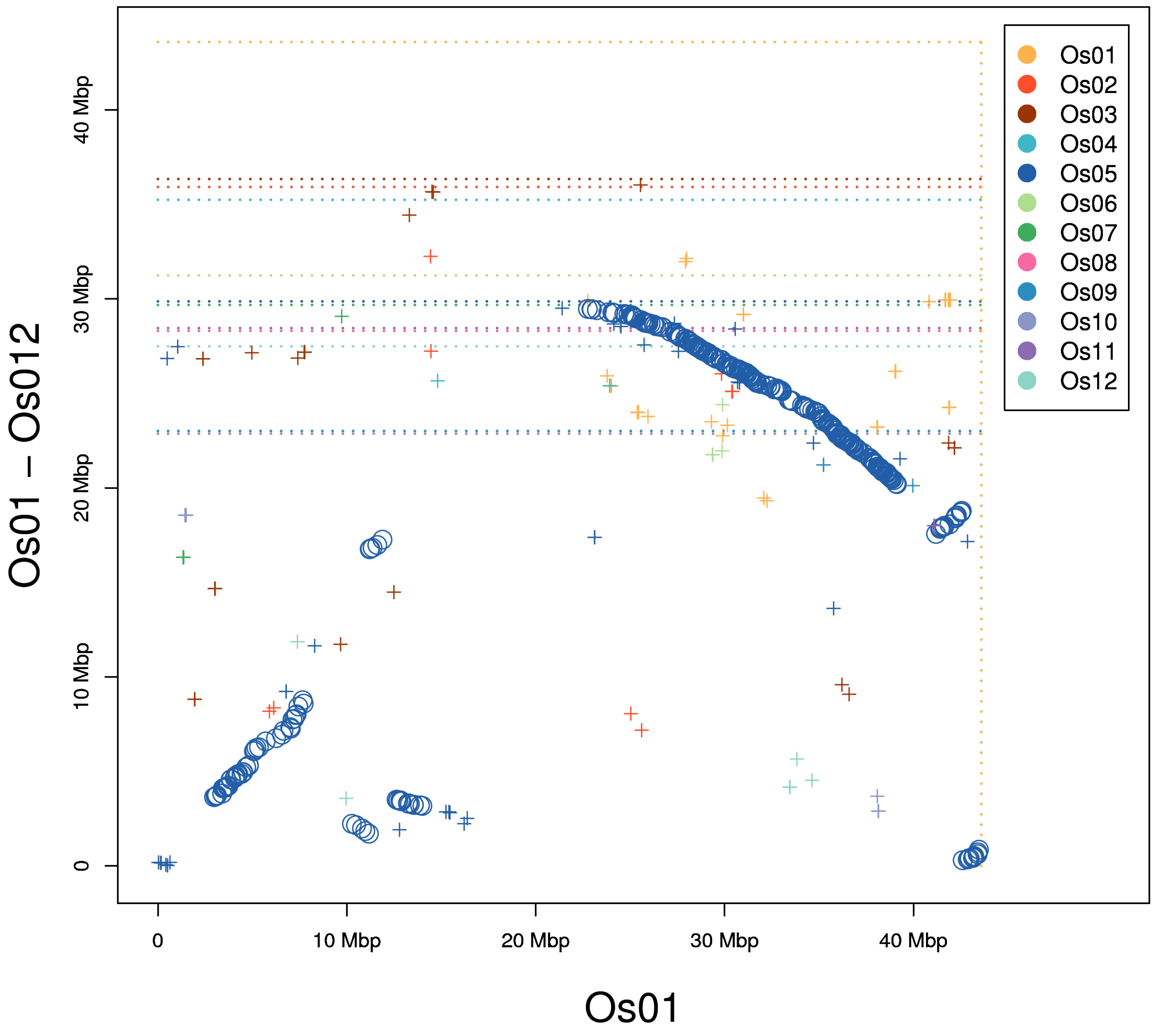

Figure S2: Duplicated segments within the rice genome. For each chromosome (Os01 – Os12), coordinates of duplicated genes located elsewhere within the rice genome, as indicated by color-codes, were plotted against each other after automatically removing local duplications and 'background' noise (see Methods). Gene pairs assigned to duplicated segments are indicated by circles.

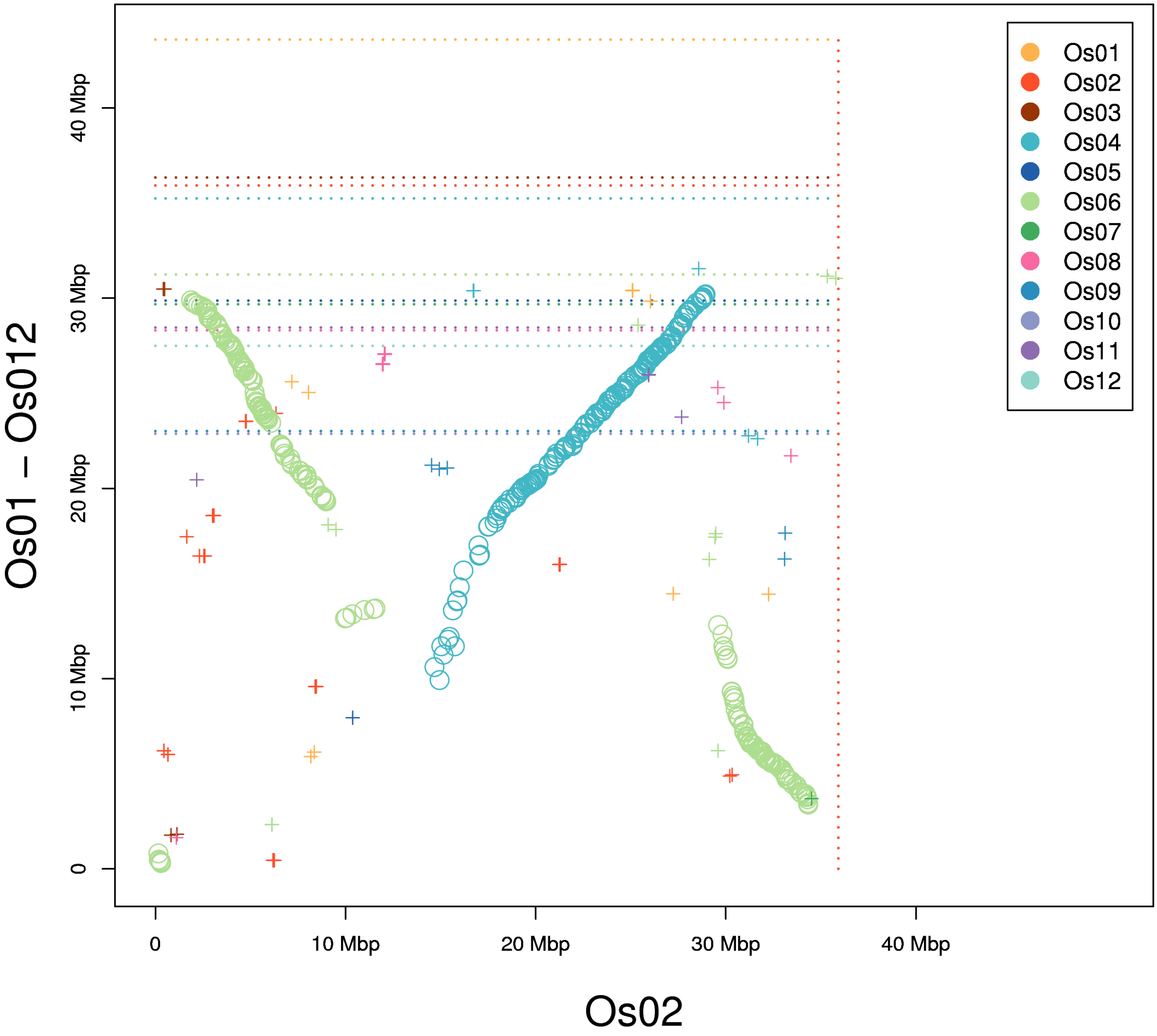

Figure S2 (continued)

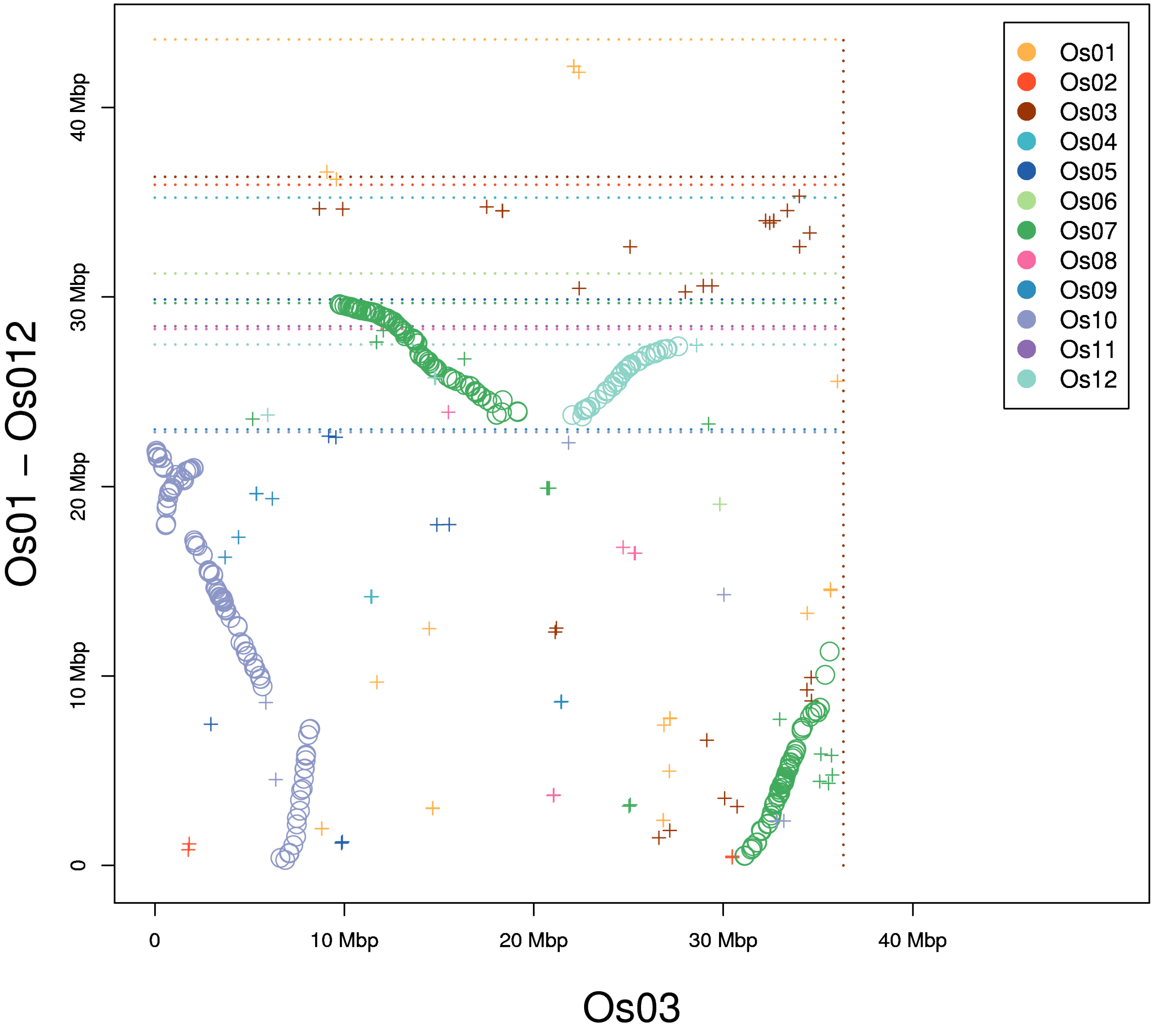

Figure S2 (continued)

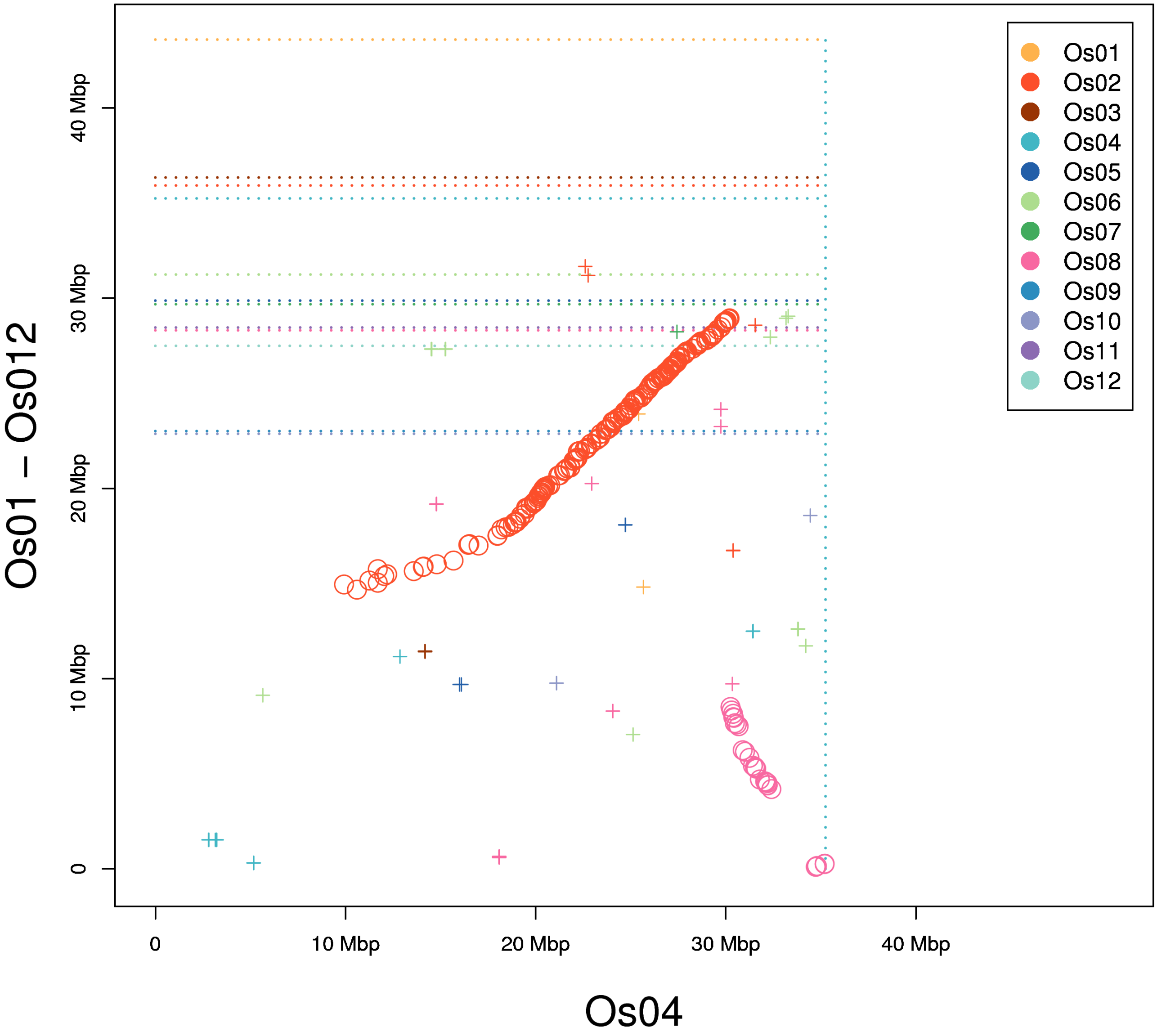

Figure S2 (continued)

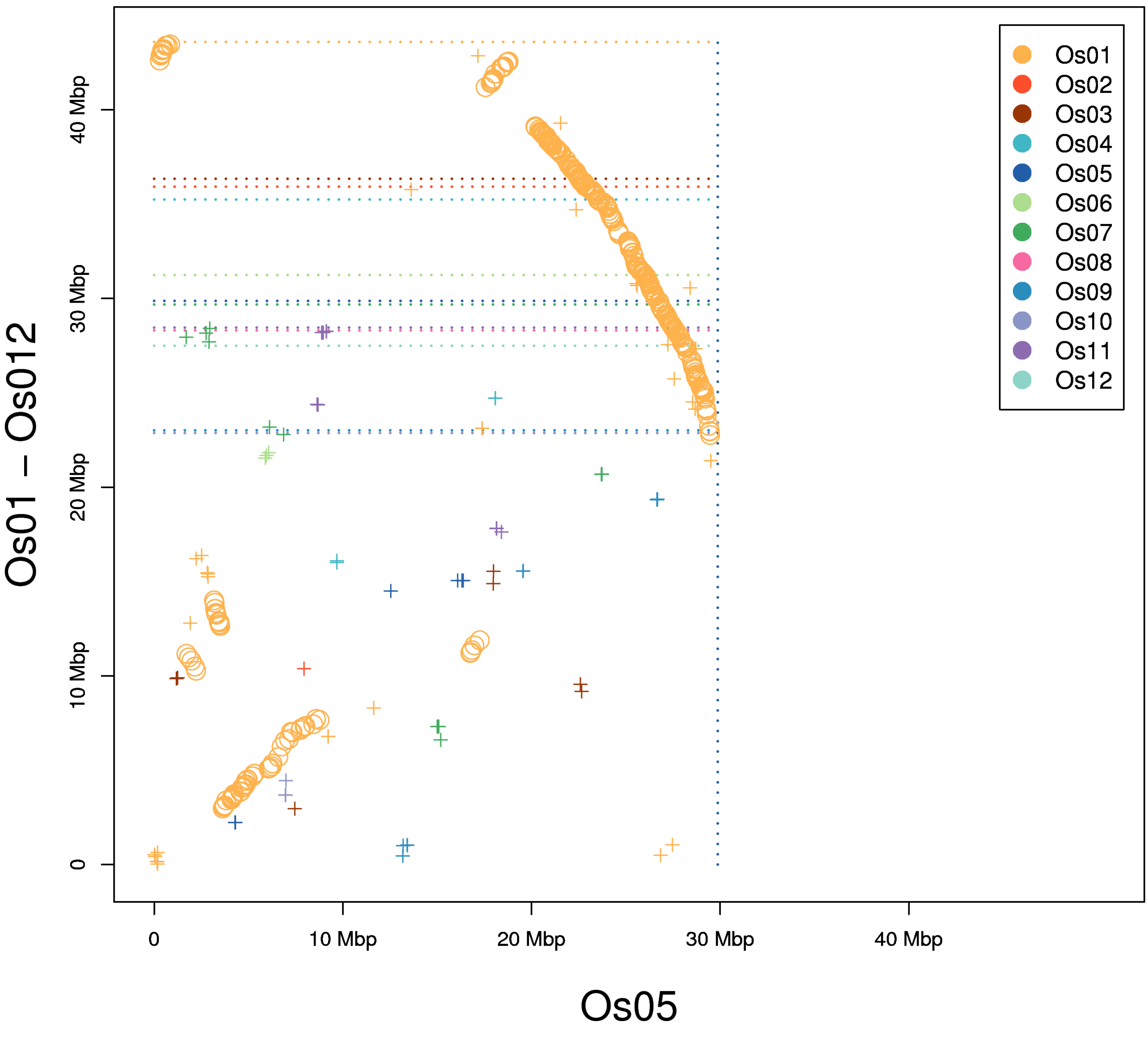

Figure S2 (continued)

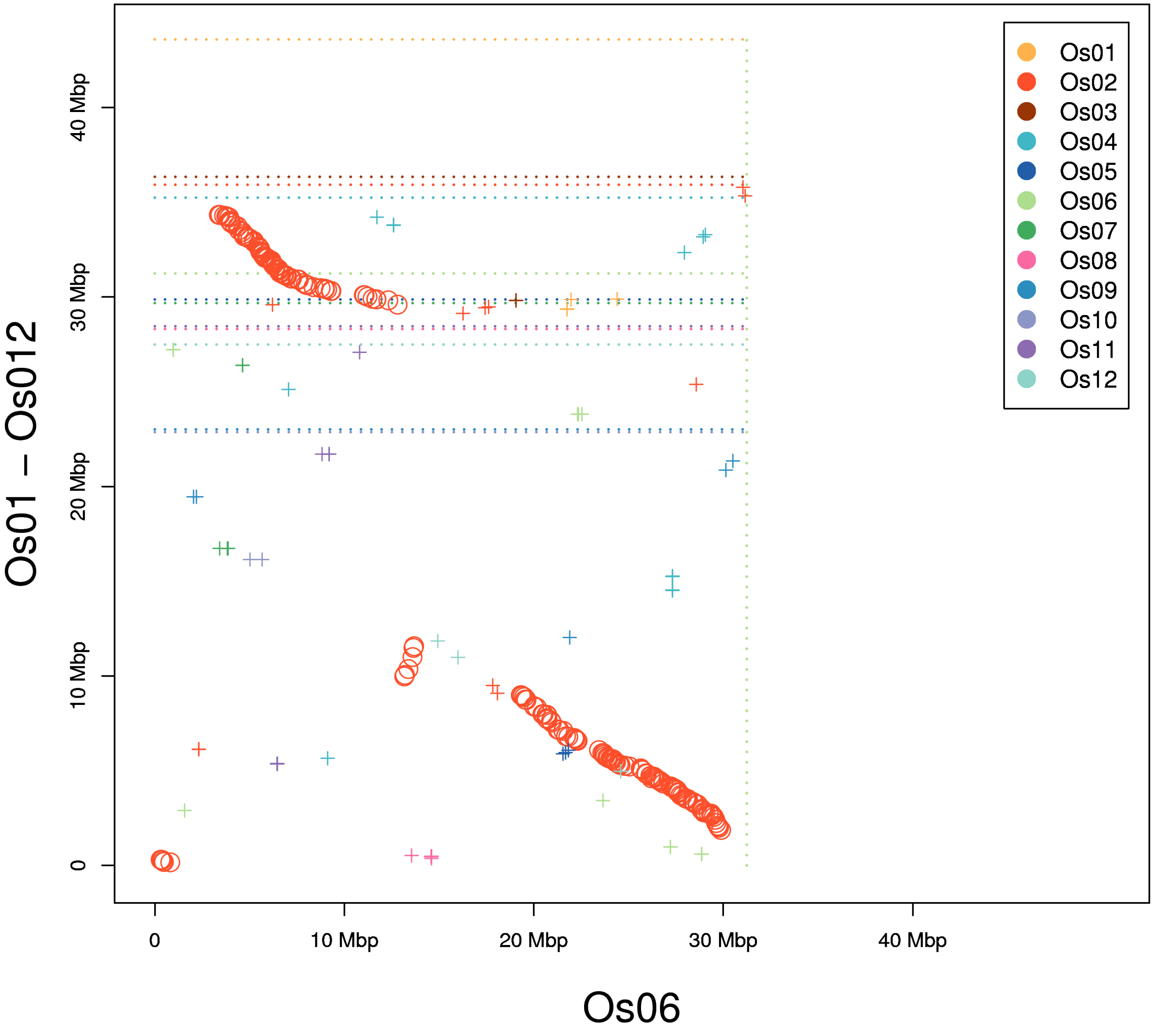

Figure S2 (continued)

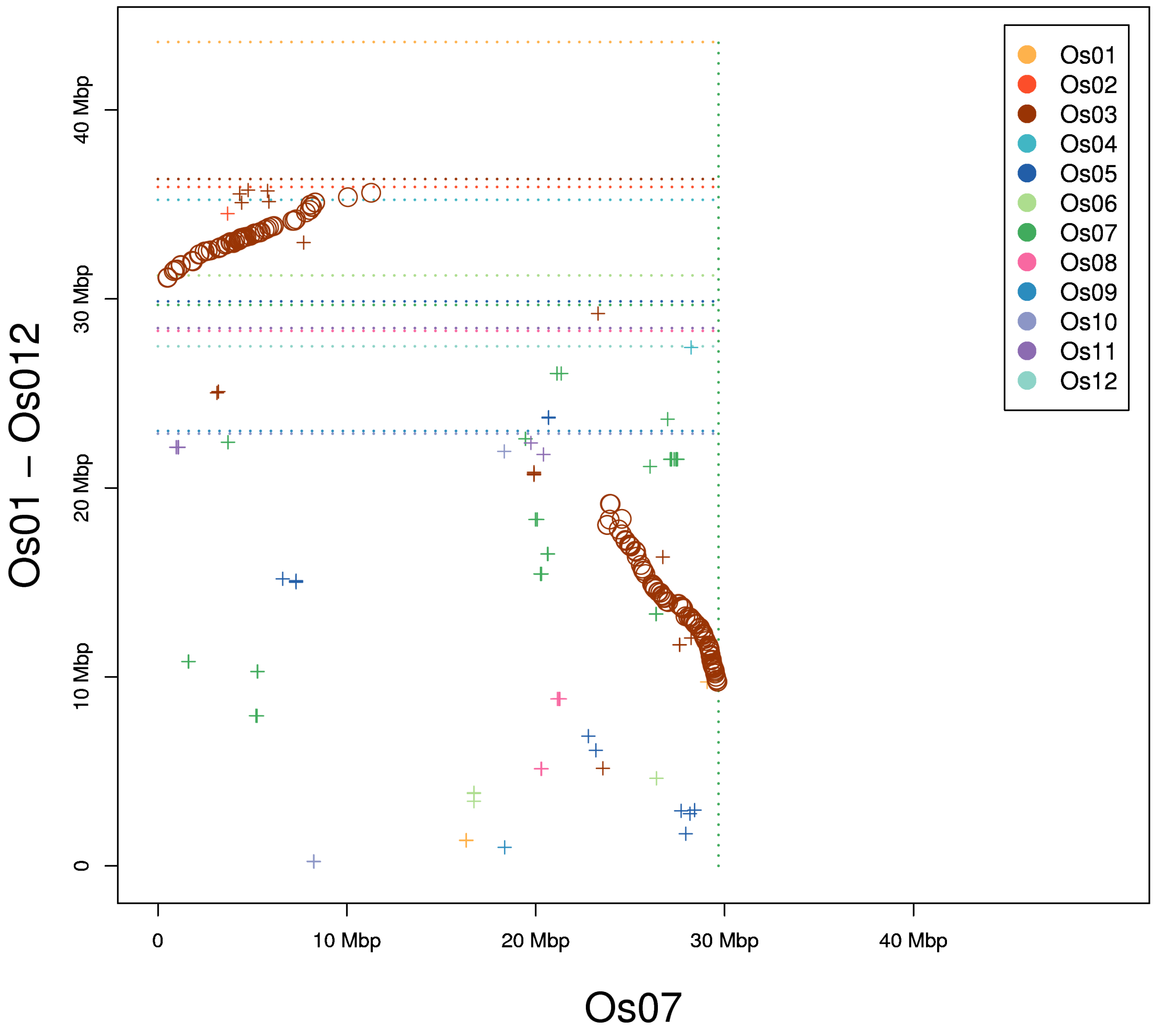

Figure S2 (continued)

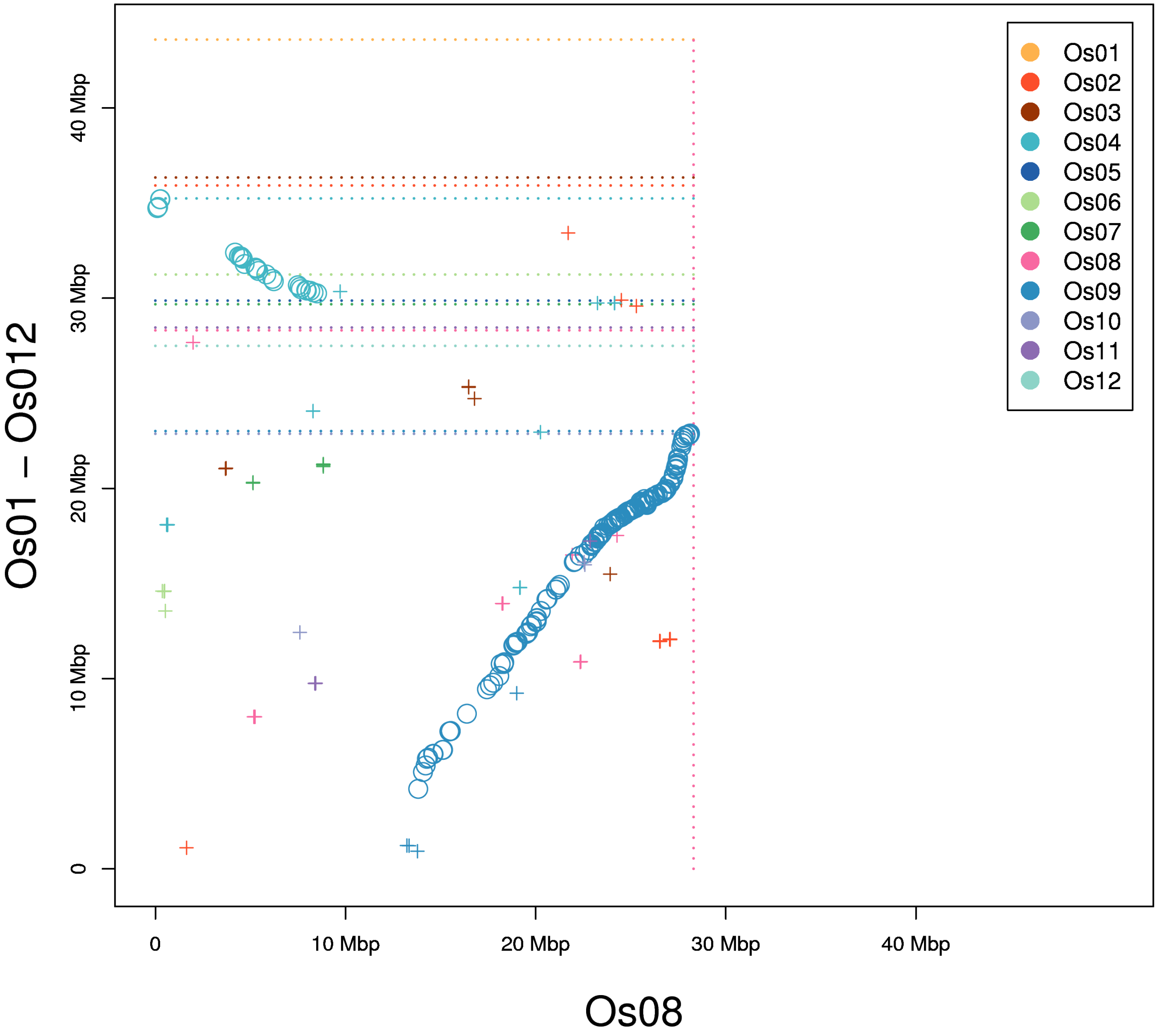

Figure S2 (continued)

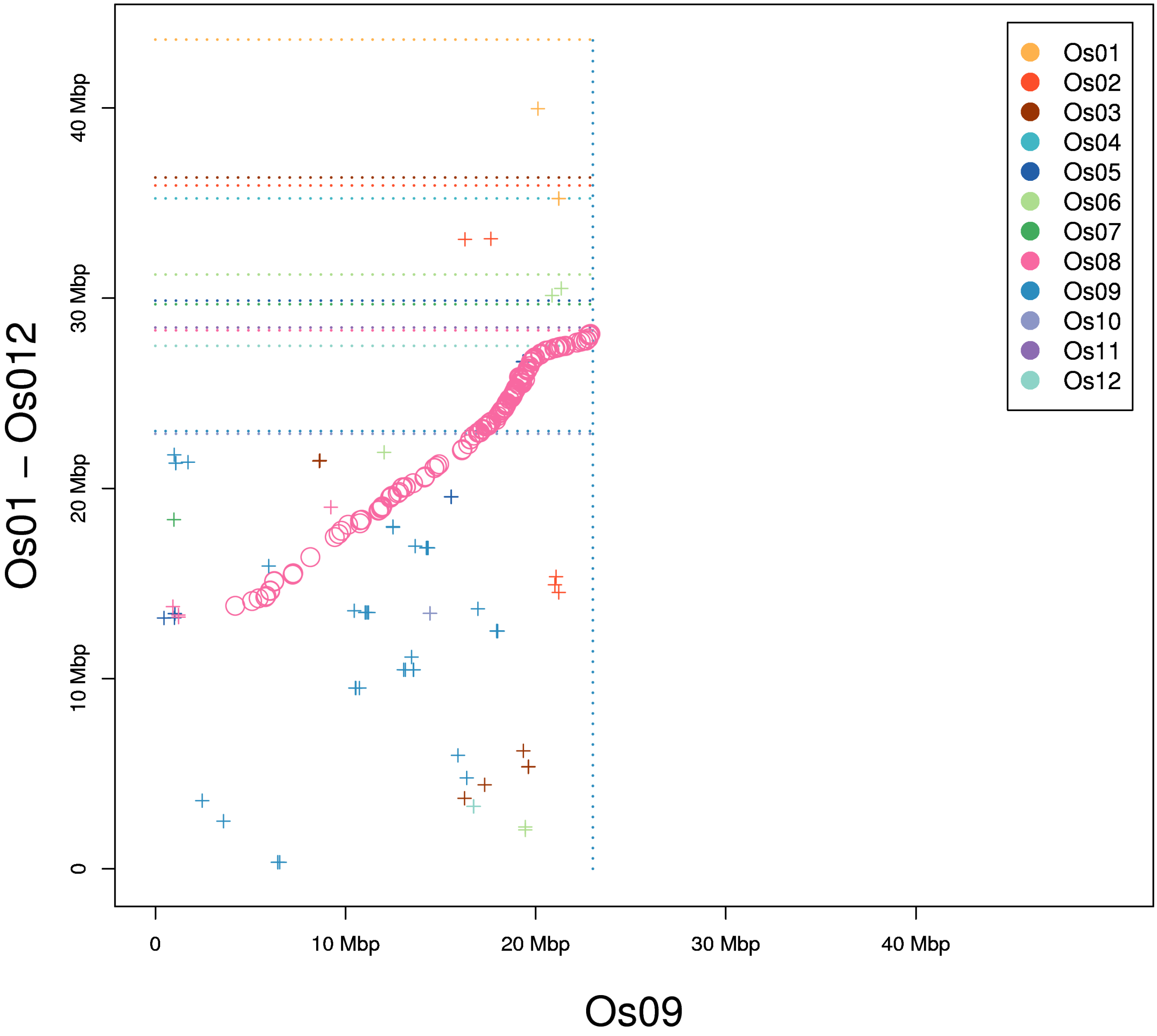

Figure S2 (continued)

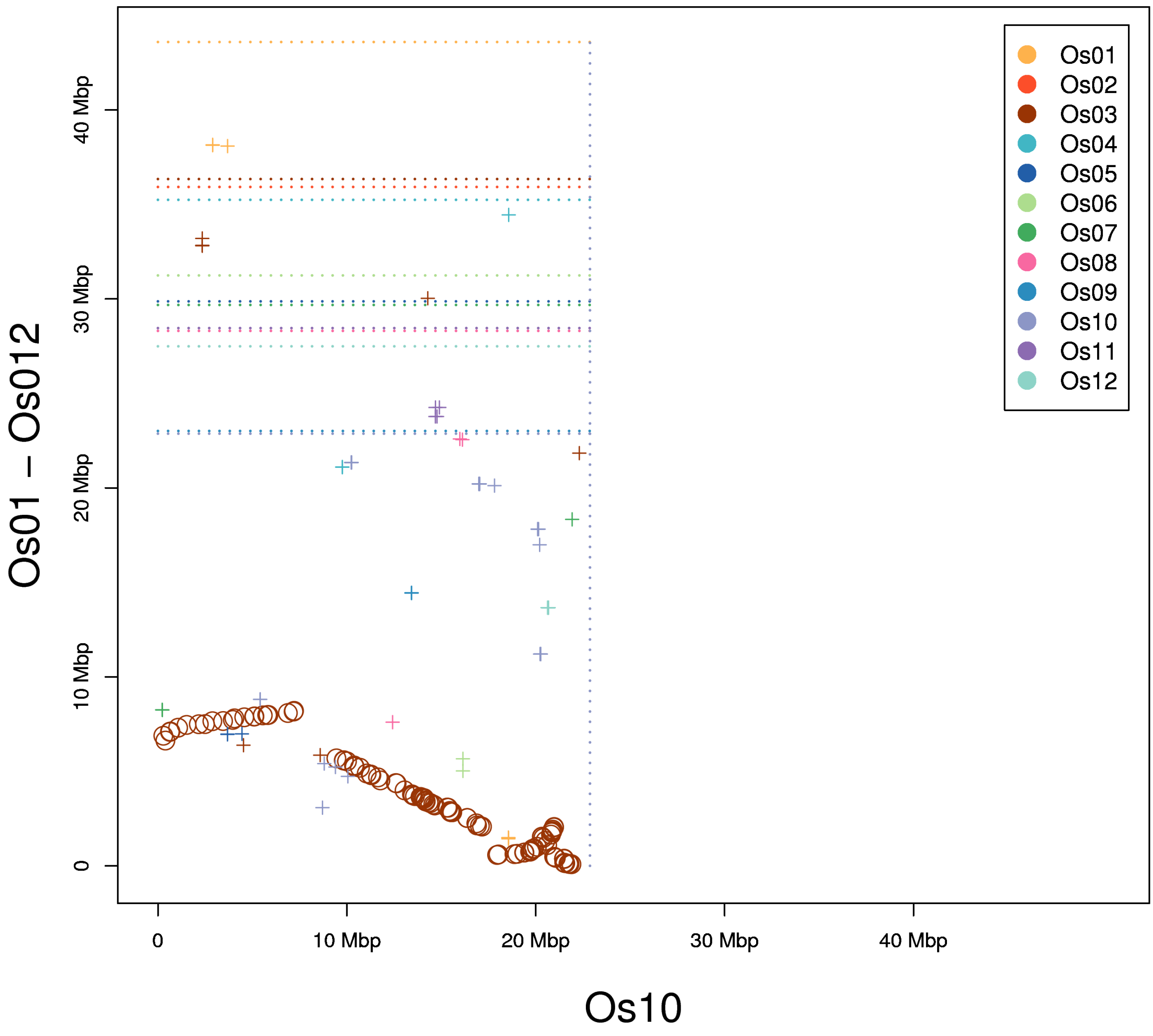

Figure S2 (continued)

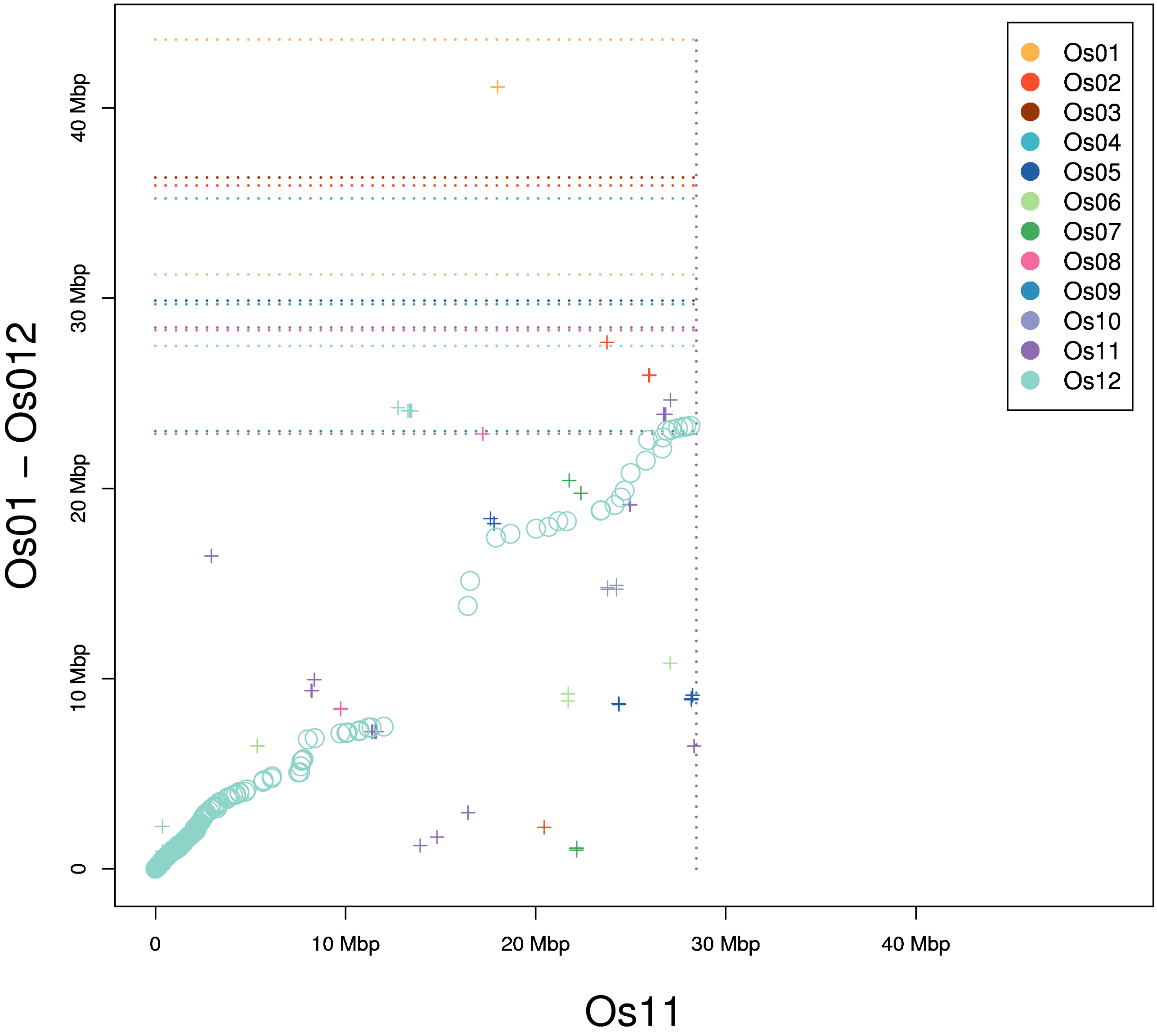

Figure S2 (continued)

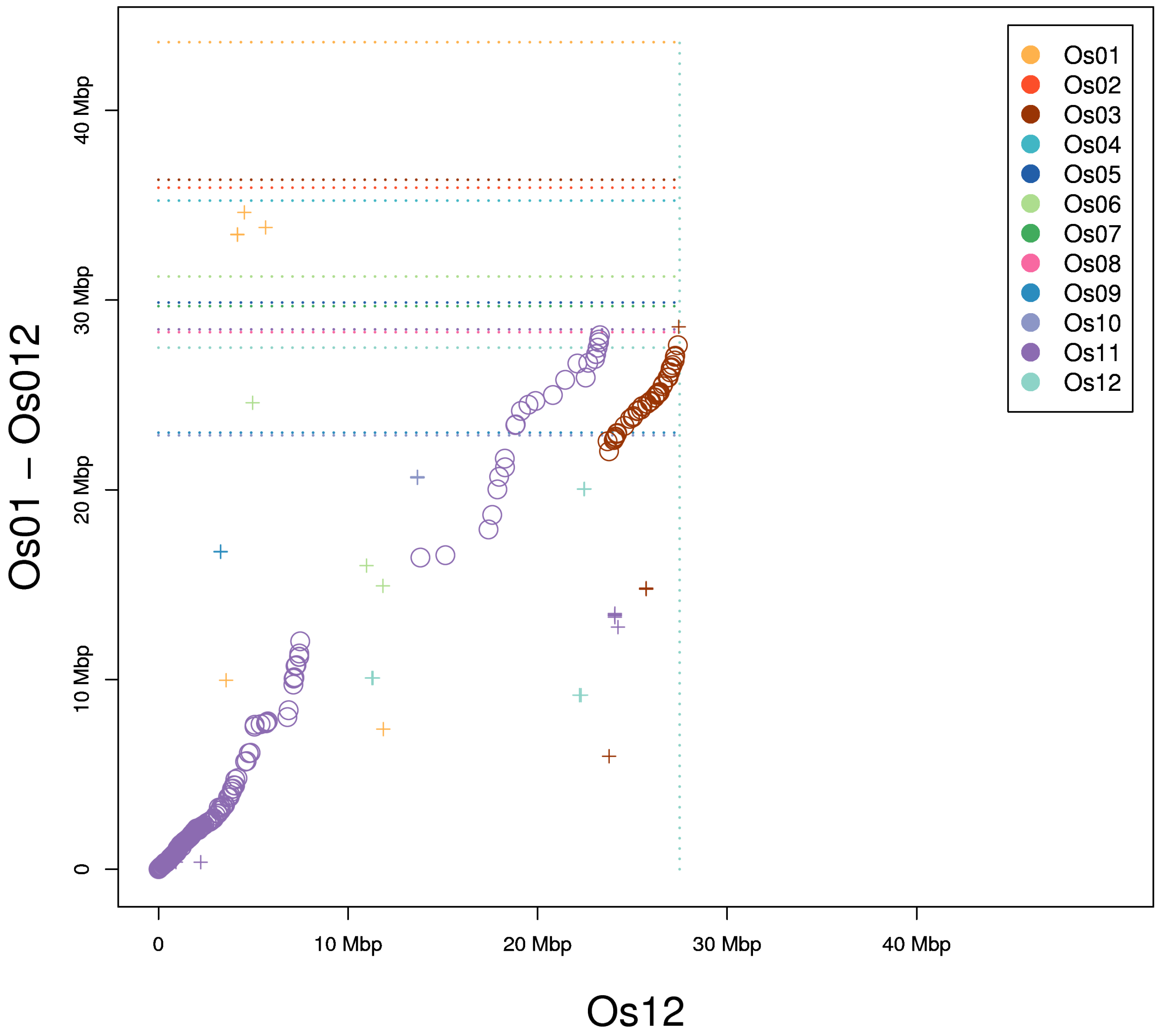

Figure S2 (continued)

Table S2: Extent of rice segmental duplications and shared synteny with barley.

	Os01	Os02	Os03	Os04	Os05	Os06	Os07	Os08	Os09	Os10	Os11	Os12	ALL
chrosomome size (kb)	43593	35925	36343	35244	29872	31243	29678	28307	23011	22872	28456	27494	372038
segmentally duplicated (kb)	25793 (59%)	28097 (78%)	26743 (74%)	22365 (63%)	17562 (59%)	21497 (69%)	17058 (57%)	18898 (67%)	18684 (81%)	18732 (82%)	24235 (85%)	21121 (77%)	260785 (70%)
not segmentally duplicated (kb)	17800 (41%)	7828 (22%)	9600 (26%)	12879 (37%)	12310 (41%)	9746 (31%)	12620 (43%)	9409 (33%)	4327 (19%)	4140 (18%)	4221 (15%)	6373 (23%)	111253 (30%)
shared synteny with barley (kb)	30521 (70%)	26023 (72%)	21432 (59%)	22594 (64%)	14923 (50%)	31061 (99%)	13137 (44%)	21546 (76%)	21611 (94%)	10123 (44%)	5161 (18%)	17714 (64%)	235846 (63%)
not syntenic to barley (kb)	13072 (30%)	9902 (28%)	14911 (41%)	12650 (36%)	14949 (50%)	182 (1%)	16541 (56%)	6761 (24%)	1400 (6%)	12749 (56%)	23295 (82%)	9780 (36%)	136192 (37%)
nr of syn markers (frequency per kb chr)	162 (1/269)	120 (1/299)	155 (1/234)	109 (1/323)	93 (1/321)	95 (1/329)	72 (1/412)	34 (1/833)	48 (1/479)	30 (1/762)	8 (1/3557)	42 (1/655)	968 (1/384)

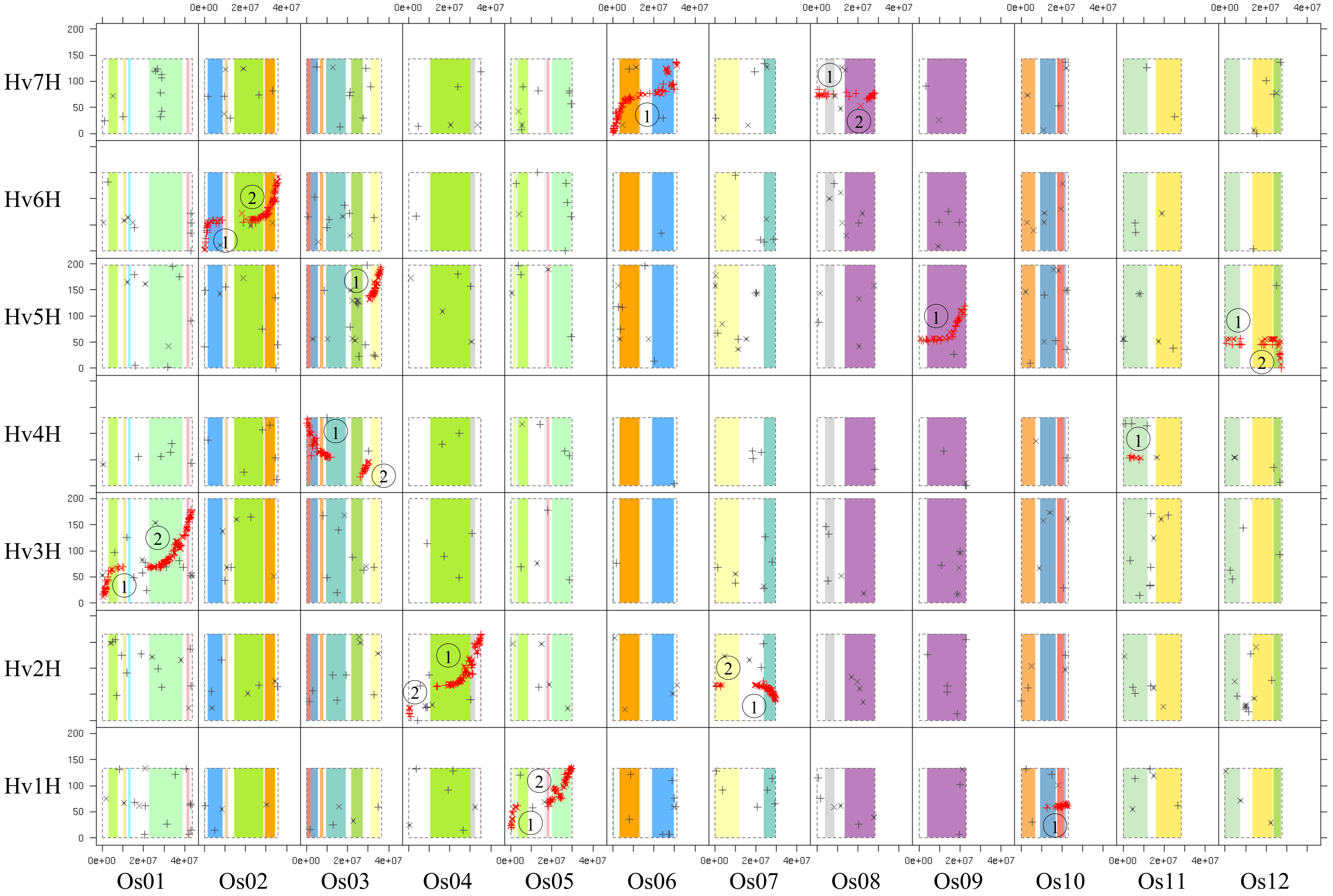

Figure S3: Shared synteny between barley and rice. Coordinates of mapped barley ESTs (chromosomes Hv1H – Hv7H) and their best rice homologs (chromosomes Os01 – Os12) are plotted against each other. Orthologs assigned to syntenic segments are shown in red, remaining homologs in grey. Numbers at segments follow the numbering schema of Table S3. Pairs of duplicated segments within the rice genome (Table S1) are indicated by matching colors of underlying filled boxes. Different sources of barley mapping data are indicated ('+' for 'map1', 'x' for 'map2', see Methods). Coordinates are in bp and cM for rice and barley, respectively.

Table S3: Shared synteny between barley and rice.

syntenic segment	barley chromo-some	barley start (cM)	barley start cDNA	barley end (cM)	barley end cDNA	rice chromo-some	rice start (Mb)	rice start TIGR locus ID	rice end (Mb)	rice end TIGR locus ID	number of orthologs	significance [1]
hv1H_os05_1	Hv1H	19.3	HZ58F06	61.2	HY07O10	Os05	0.1	Os05g01240	3.3	Os05g06500	17	< 2.2E-16
hv1H_os05_2	Hv1H	60.9	HW02D17	133.9	HY06P08	Os05	17.9	Os05g31000	29.7	Os05g51830	76	< 2.2E-16
hv1H_os10_1	Hv1H	58.1	HY04D20	65.3	32_8486	Os10	12.6	Os10g25130	22.8	Os10g42820	30	< 2.2E-16
hv2H_os04_1	Hv2H	64.7	HH01F06	165.1	MWG949a	Os04	13.6	Os04g23830	35.2	Os04g59610	99	< 2.2E-16
hv2H_os04_2	Hv2H	7.2	HW08K11	25.1	32_3627	Os04	0.2	Os04g01230	1.1	Os04g02900	10	< 2.2E-16
hv2H_os07_1	Hv2H	37.7	HE01A24	75.2	MWG2211	Os07	19.6	Os07g32800	29.6	Os07g49400	65	< 2.2E-16
hv2H_os07_2	Hv2H	65.1	HW01M06	69.6	32_6510	Os07	0.3	Os07g01490	3.5	Os07g07060	7	1.5E-08
hv3H_os01_1	Hv3H	12.2	32_1173	68.9	HK05A23	Os01	0.4	Os01g01790	9.9	Os01g17240	36	< 2.2E-16
hv3H_os01_2	Hv3H	68.8	HY03N03	178.7	HW05M08	Os01	22.3	Os01g39110	43.3	Os01g74250	126	< 2.2E-16
hv4H_os03_1	Hv4H	53.9	HV05N05	127.3	HA10C05	Os03	0.1	Os03g01200	11.8	Os03g20780	62	< 2.2E-16
hv4H_os03_2	Hv4H	16.6	HY04F13	45.9	32_14765	Os03	26.2	Os03g46390	30.3	Os03g52840	28	< 2.2E-16
hv4H_os11_1	Hv4H	50.3	HY02N17	57.1	HW04N08	Os11	3.3	Os11g06720	8.4	Os11g14980	8	3.5E-10
hv5H_os03_1	Hv5H	131.8	32_1863	193.6	HY05G12	Os03	30.3	Os03g53000	36.1	Os03g63950	65	< 2.2E-16
hv5H_os09_1	Hv5H	51.7	HY02O02	119.8	HY04C22	Os09	0.7	Os09g01960	22.3	Os09g38750	48	< 2.2E-16
hv5H_os12_1	Hv5H	45.2	HK03K09	56.1	HW04N22	Os12	0.6	Os12g02094	8.0	Os12g14070	10	2.3E-11
hv5H_os12_2	Hv5H	0.0	HW05F15	55.8	32_8631	Os12	17.6	Os12g29580	27.4	Os12g44150	32	< 2.2E-16
hv6H_os02_1	Hv6H	1.6	32_1692	60.4	32_9863	Os02	0.0	Os02g01070	8.5	Os02g15220	30	< 2.2E-16
hv6H_os02_2	Hv6H	53.1	HY10K15	141.4	32_3363	Os02	18.2	Os02g30630	35.8	Os02g58560	90	< 2.2E-16
hv7H_os06_1	Hv7H	2.7	HY07N21	136.7	HY03C01	Os06	0.1	Os06g01210	31.2	Os06g51510	95	< 2.2E-16
hv7H_os08_1	Hv7H	71.2	HY02C18	84.3	HY03F15	Os08	0.2	Os08g01370	7.9	Os08g13350	13	3.9E-15
hv7H_os08_2	Hv7H	52.8	32_11387	78.8	HW01L11	Os08	14.3	Os08g23810	28.1	Os08g45010	21	< 2.2E-16

[1] one-sided Fisher test, see Methods for details

Table S4: Shared ancestral duplications between barley and rice.

	barley					rice						number of homologs [2]			
	chromo-	start [1]		end [1]		chromo-	start [1]		end [1]		segmental	orthologous	paralogous	Fisher's test	syntenic
block	some	(cM)	cDNA	(cM)	cDNA	some	(Mb)	TIGR locus ID	(Mb)	TIGR locus ID	duplication	segment	segment	p-value	segment
AD_1	Hv1H	72.8	32_7299	133.1	32_4927	Os05	20.4	Os05g34540	29.5	Os05g51530	os01_05_5	63 (2)	3 (24)	1.1E-16	hv1H_os05_2
AD_1	Hv3H	68.8	HY03L07	127.7	32_3965	Os01	23.1	Os01g40310	38.9	Os01g66330	os01_05_5	92 (3)	1 (28)	2.5E-23	hv3H_os01_2
AD_2	Hv6H	52.1	HY05I23	60.4	32_9863	Os02	2.0	Os02g04460	8.5	Os02g15220	os02_06_2	9 (0)	1 (2)	4.5E-02	hv6H_os02_1
AD_2	Hv7H	74.9	32_3232	124.5	32_5507	Os06	21.0	Os06g35970	29.5	Os06g48750	os02_06_2	32 (3)	6 (12)	2.0E-05	hv7H_os06_2
AD_3	Hv2H	64.7	HH01F06	119.7	32_7576	Os04	13.6	Os04g23830	30.0	Os04g50990	os02_04_1	58 (10)	7 (25)	9.4E-10	hv2H_os04_1
AD_3	Hv6H	53.1	HY10K15	71.6	32_4064	Os02	18.2	Os02g30630	28.8	Os02g47220	os02_04_1	30 (2)	2 (19)	3.3E-10	hv6H_os02_2
AD_4	Hv6H	65.6	HI04I03	113.3	HY05G09	Os02	29.7	Os02g48560	34.1	Os02g55640	os02_06_4	40 (0)	0 (9)	4.9E-10	hv6H_os02_2
AD_4	Hv7H	41.3	HY03P21	69.9	HY02G19	Os06	3.4	Os06g07120	9.1	Os06g15990	os02_06_4	23 (2)	1 (8)	2.1E-05	hv7H_os06_1
AD_5	Hv1H	58.1	HY04D20	64.3	32_1955	Os10	12.6	Os10g25130	21.6	Os10g40810	os03_10 [3]	21 (2)	1 (6)	3.1E-04	hv1H_os10_1
AD_5	Hv4H	57.6	HY05N11	127.3	HA10C05	Os03	0.1	Os03g01200	5.5	Os03g10800	os03_10 [3]	30 (1)	3 (7)	3.9E-05	hv4H_os03_1
AD_6	Hv5H	51.7	HY02O02	119.8	HY04C22	Os09	4.4	Os09g08430	22.3	Os09g38750	os08_09_1	43 (3)	2 (18)	3.3E-11	hv5H_os09_1
AD_6	Hv7H	52.8	32_11387	78.8	HW01L11	Os08	14.3	Os08g23810	28.1	Os08g45010	os08_09_1	21 (0)	1 (13)	9.5E-09	hv7H_os08_2
AD_7	Hv2H	37.7	HE01A24	66.4	HY09N16	Os07	24.5	Os07g40890	29.6	Os07g49400	os03_07_1	48 (5)	6 (18)	1.6E-08	hv2H_os07_1
AD_7	Hv4H	53.9	HV05N05	57.1	HW02O04a	Os03	9.8	Os03g17690	11.8	Os03g20780	os03_07_1	12 (0)	0 (7)	2.0E-05	hv4H_os03_1

[1] names and coordinates of the marginal barley and rice orthologs of corresponding segments are given

[2] numbers of ‘best’ and ‘second-best’ (in brackets) homologs of barley ESTs are given for both the corresponding orthologous and paralogous segments within the rice genome

[3] three contiguous but inverted rice duplications (os03_10_1, os03_10_2, os03_10_3) were summarized to AD_5

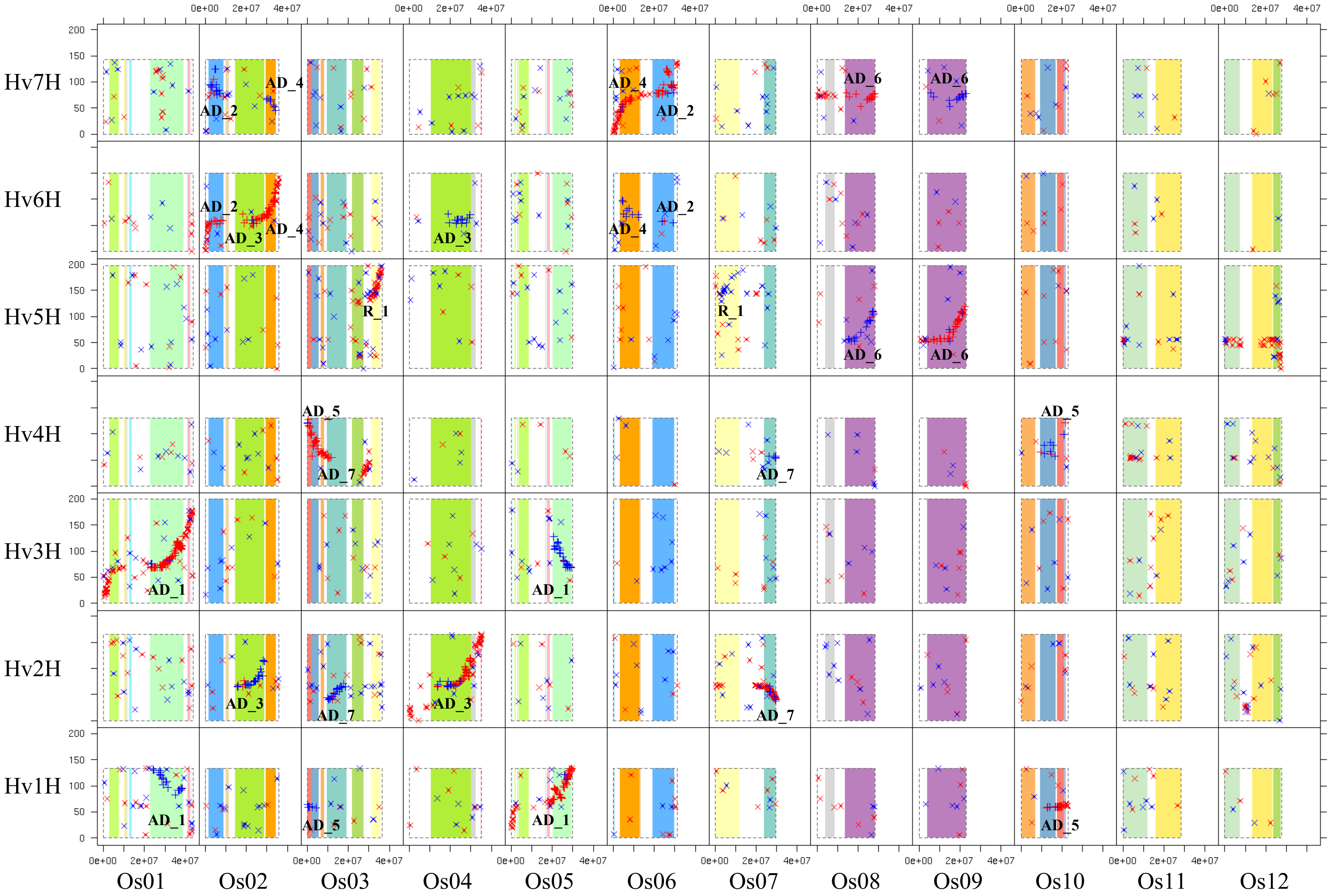

Figure S4: Shared ancestral duplications between barley and rice. Coordinates of mapped barley ESTs (Hv1H – Hv7H) are plotted against best (red) and second-best (blue), non-locally duplicated, rice homologs (Os01 – Os12). Membership to ancestral duplicated blocks (AD_1 – AD_7) is indicated by '+' symbols (otherwise by 'x'). Note the accordance of the distribution of best and second-best rice homologs and the location of duplicated rice segments (matching colors of underlying filled boxes, see Table S1). R_1 corresponds to a barley segment on Hv5H with orthology to Os03 and paralogy to Os07. Coordinates are in bp and cM for rice and barley, respectively.

Table S5: Positive correlation of shared synteny with barley and retention of WGD paralogs in rice.

ancestral duplicated block	ortholog pair		rice gene is duplicated	rice gene not duplicated	total	ratio duplicated versus not duplicated	odds ratio	one-sided Fisher test p-value
AD_1	Hv1H / Os05	ortholog in barley found	24	39	63	**0.62**	**2.19**	3.2E-03
		orthology to barley unknown	330	1177	1507	**0.28**		
		total	354	1216	1570	0.29		
	Hv3H / Os01	ortholog in barley found	26	66	92	**0.39**	**2.50**	2.5E-04
		orthology to barley unknown	328	2081	2409	**0.16**		
		total	354	2147	2501	0.16		
AD_3	Hv2H / Os04	ortholog in barley found	24	34	58	**0.71**	**6.99**	1.5E-10
		orthology to barley unknown	264	2613	2877	**0.10**		
		total	288	2647	2935	0.11		
	Hv6H / Os02	ortholog in barley found	15	15	30	**1.00**	**6.57**	1.8E-06
		orthology to barley unknown	273	1794	2067	**0.15**		
		total	288	1809	2097	0.16		
AD_6	Hv5H / Os09	ortholog in barley found	16	27	43	**0.59**	**7.15**	7.5E-08
		orthology to barley unknown	212	2559	2771	**0.08**		
		total	228	2586	2814	0.09		
	Hv7H / Os08	ortholog in barley found	10	11	21	**0.91**	**7.84**	2.1E-05
		orthology to barley unknown	218	1881	2099	**0.12**		
		total	228	1892	2120	0.12		

Table S6: Comparison of location and extent of rice segmental duplications to previous studies [11, 12, 19].

	Os01	Os02	Os03	Os04	Os05	Os06	Os07	Os08	Os09	Os10	Os11	Os12	ALL
chrosomome size (kb)	43593	35925	36343	35244	29872	31243	29678	28307	23011	22872	28456	27494	372038
**total duplicated segment size (kb)**	**25793 (59%)**	**28097 (78%)**	**26743 (74%)**	**22365 (63%)**	**17562 (59%)**	**21497 (69%)**	**17058 (57%)**	**18898 (67%)**	**18684 (81%)**	**18732 (82%)**	**24235 (85%)**	**21121 (77%)**	**260785 (70%)**
Yu *et al.* [11] total (relative to this study)	31000 (120%)	28800 (103%)	23500 (88%)	23200 (104%)	19800 (113%)	25300 (118%)	13900 (81%)	17200 (91%)	16300 (87%)	19700 (105%)	13900 (57%)	13300 (63%)	245900 (94%)
overlap with this study	20800 (81%)	25204 (90%)	13959 (52%)	20685 (92%)	13045 (74%)	20160 (94%)	12574 (74%)	14706 (78%)	15999 (86%)	16937 (90%)	13273 (55%)	13300 (63%)	200642 (77%)
no overlap, only identified in this study	4993 (19%)	2893 (10%)	12784 (48%)	1680 (8%)	4517 (26%)	1337 (6%)	4484 (26%)	4192 (22%)	2685 (14%)	1795 (10%)	10962 (45%)	7821 (37%)	60143 (23%)
no overlap, only identified by Yu *et al.* [11]	10200 (33%)	3596 (12%)	9541 (41%)	2515 (11%)	6755 (34%)	5140 (20%)	1326 (10%)	2494 (15%)	301 (2%)	2763 (14%)	627 (5%)	0 (0%)	45258 (18%)
Wang *et al.* [12] total (relative to this study)	20880 (81%)	26860 (96%)	16110 (60%)	14920 (67%)	14170 (81%)	20750 (97%)	13810 (81%)	10370 (55%)	11560 (62%)	6310 (34%)	5440 (22%)	4270 (20%)	165450 (63%)
Salse *et al.* [19] total (relative to this study)	28160 (109%)	31910 (114%)	31340 (117%)	14580 (65%)	20060 (114%)	26170 (122%)	21960 (129%)	23910 (127%)	20180 (108%)	12070 (64%)	23140 (95%)	18230 (86%)	271710 (104%)
overlap with this study	20596 (80%)	27204 (97%)	25805 (96%)	12866 (58%)	12920 (74%)	20378 (95%)	15353 (90%)	15953 (84%)	18009 (96%)	11547 (62%)	19119 (79%)	12425 (59%)	212175 (81%)
no overlap, only identified in this study	5197 (20%)	893 (3%)	938 (4%)	9499 (42%)	4642 (26%)	1119 (5%)	1705 (10%)	2945 (16%)	675 (4%)	7185 (38%)	5116 (21%)	8696 (41%)	48610 (19%)
no overlap, only identified by Salse *et al.* [19]	7564 (24%)	4706 (16%)	5535 (24%)	1714 (7%)	7140 (36%)	5792 (23%)	6607 (48%)	7957 (46%)	2171 (13%)	523 (3%)	4021 (29%)	5805 (44%)	59535 (24%)
Salse *et al.* [19] incl superimposed segments	40010	64780	43360	18610	47570	46940	38490	37420	37610	18250	34460	33080	
Salse *et al.* [19], 2 times superimposed	6250 (22%)	11312 (35%)	12020 (38%)	4030 (28%)	2380 (12%)	13310 (51%)	14470 (66%)	12750 (53%)	14750 (73%)	6180 (51%)	11120 (48%)	8470 (46%)	117042 (43%)
Salse *et al.* [19], 3 times superimposed	2800 (10%)	5649 (18%)	0 (0%)	0 (0%)	5410 (27%)	3730 (14%)	1030 (5%)	380 (2%)	1340 (7%)	0 (0%)	100 (0%)	3190 (17%)	23629 (9%)
Salse *et al.* [19], 4 times superimposed	0 (0%)	3420 (11%)	0 (0%)	0 (0%)	4770 (24%)	0 (0%)	0 (0%)	0 (0%)	0 (0%)	0 (0%)	0 (0%)	0 (0%)	8190 (3%)

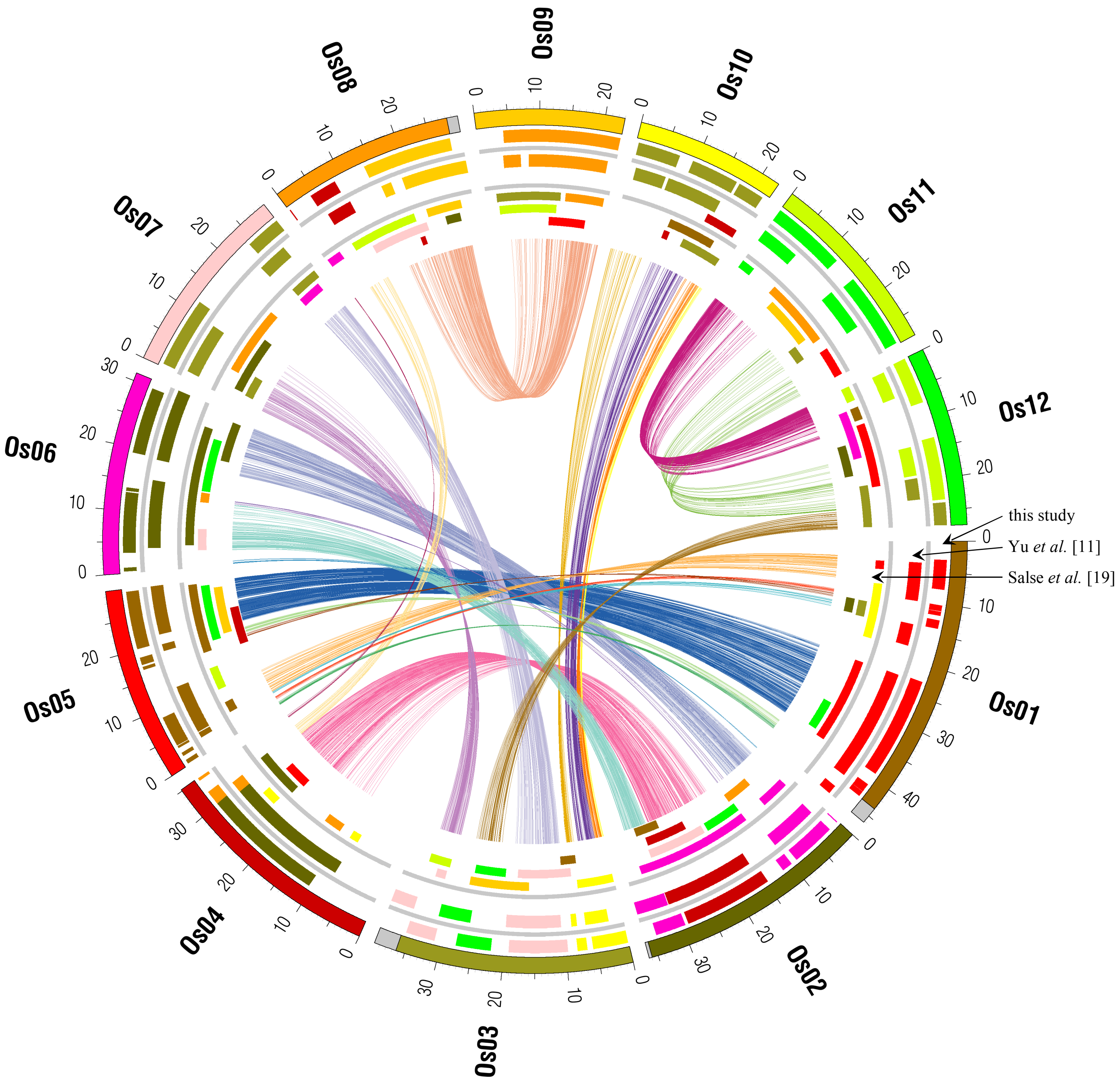

Figure S5: Comparison of location and extent of segmental duplications within the rice genome to two previous studies [11, 19]. Segmentally duplicated rice genes identified in this study are displayed by connecting lines among the 12 rice chromosomes (Os01 – Os12) and could be organized in 24 pairs of duplicated genome segments (see Figure 1). Bar colors indicate on which chromosome the corresponding duplicated segment is located, from the outer to the inner lane (separated by grey lines) as follows: (1) this study, (2) Yu *et al.* [11], (3) Salse *et al.* [19]. Note the shifted location of corresponding segments compared to Yu et al. [11] using the rice *indica* assembly.

Table S7: Mean prediction errors of regression methods used for barley map integration.

chromosome (nr of anchors)	Linear regression	Local polynomial regression	Local polynomial regression (robust)
1H (13)	2.5	1.1*	1.4
2H (29)	4.6	3.4	2.1*
3H (14)	2.3	1.1	1.0*
4H (13)	4.2	4.1	2.3*
5H (21)	4.5	3.6	3.5*
6H (22)	4.0	3.9	2.3*
7H (19)	4.9	4.6	2.8*

Mean values of prediction errors (in cM) were computed chromosome-wise for all n common EST markers between both data sets ('map1' and 'map2', see Methods) using the n-1 smallest absolute differences of the real versus the predicted mapping position thus allowing one outlier per chromosome. Asterisks show the lowest prediction error among all three regression methods per chromosome. Numbers in brackets show the number of anchor markers between both maps.

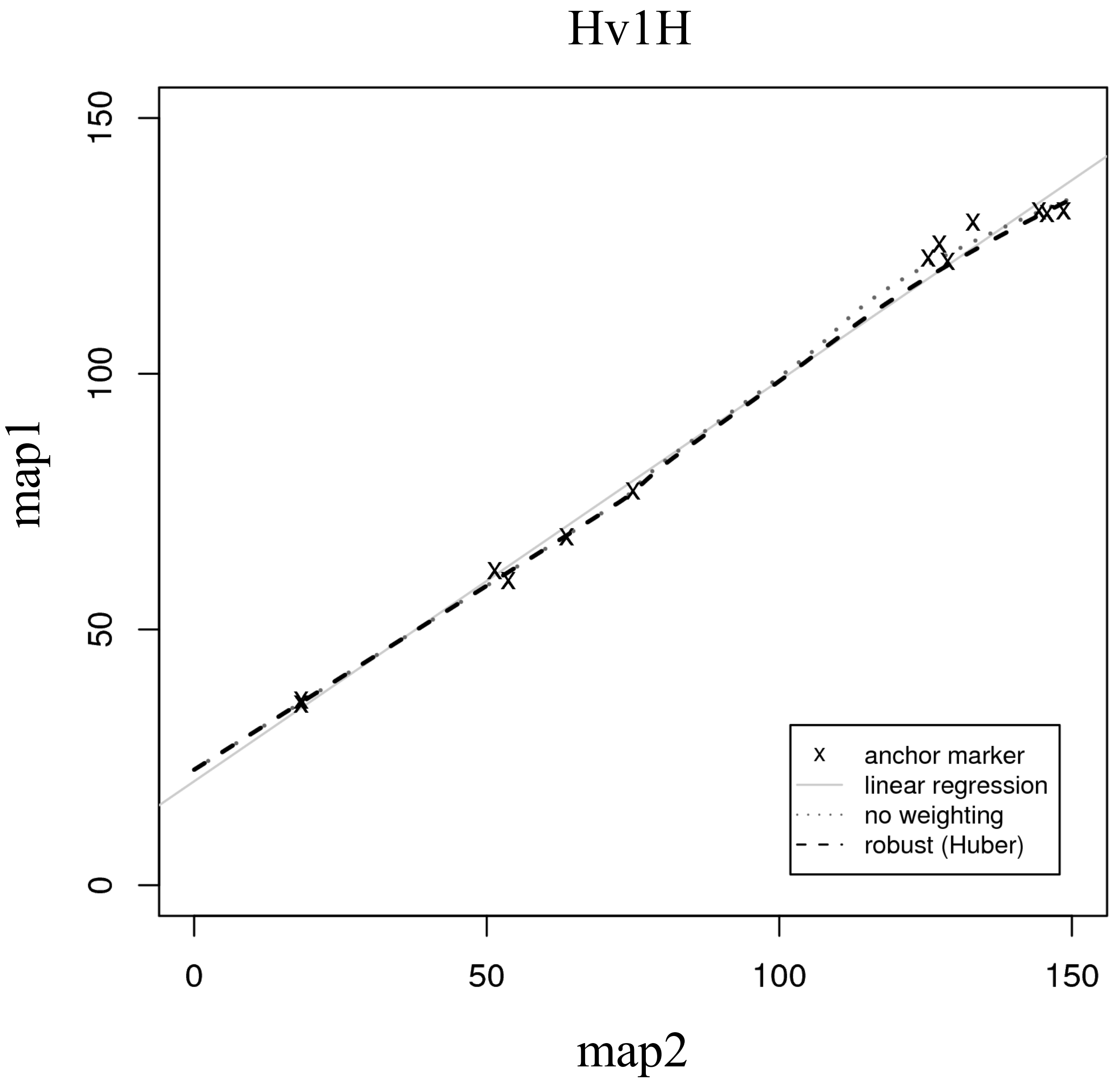

Figure S6: Regression plots for the integration of two barley genetic maps. Coordinates of common markers (chromosomes Hv1H – Hv7H) of both genetic maps (‘map1’ and ‘map2’, see Methods) were plotted against each other and used as anchor points for map integration using three regression methods (linear regression, local polynomial regression – no weighting, local polynomial regression – robust, see Methods).

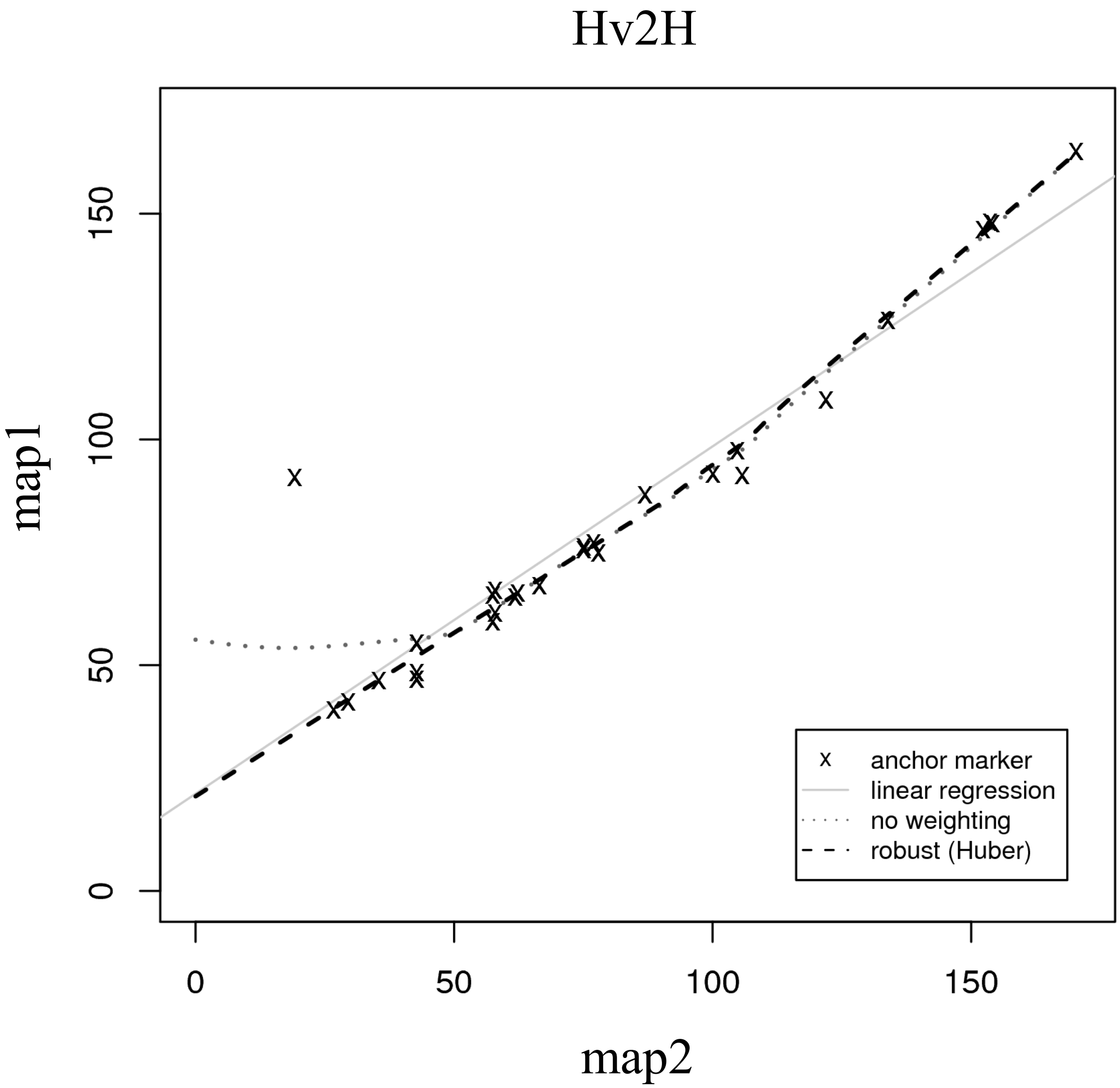

Figure S6 (continued)

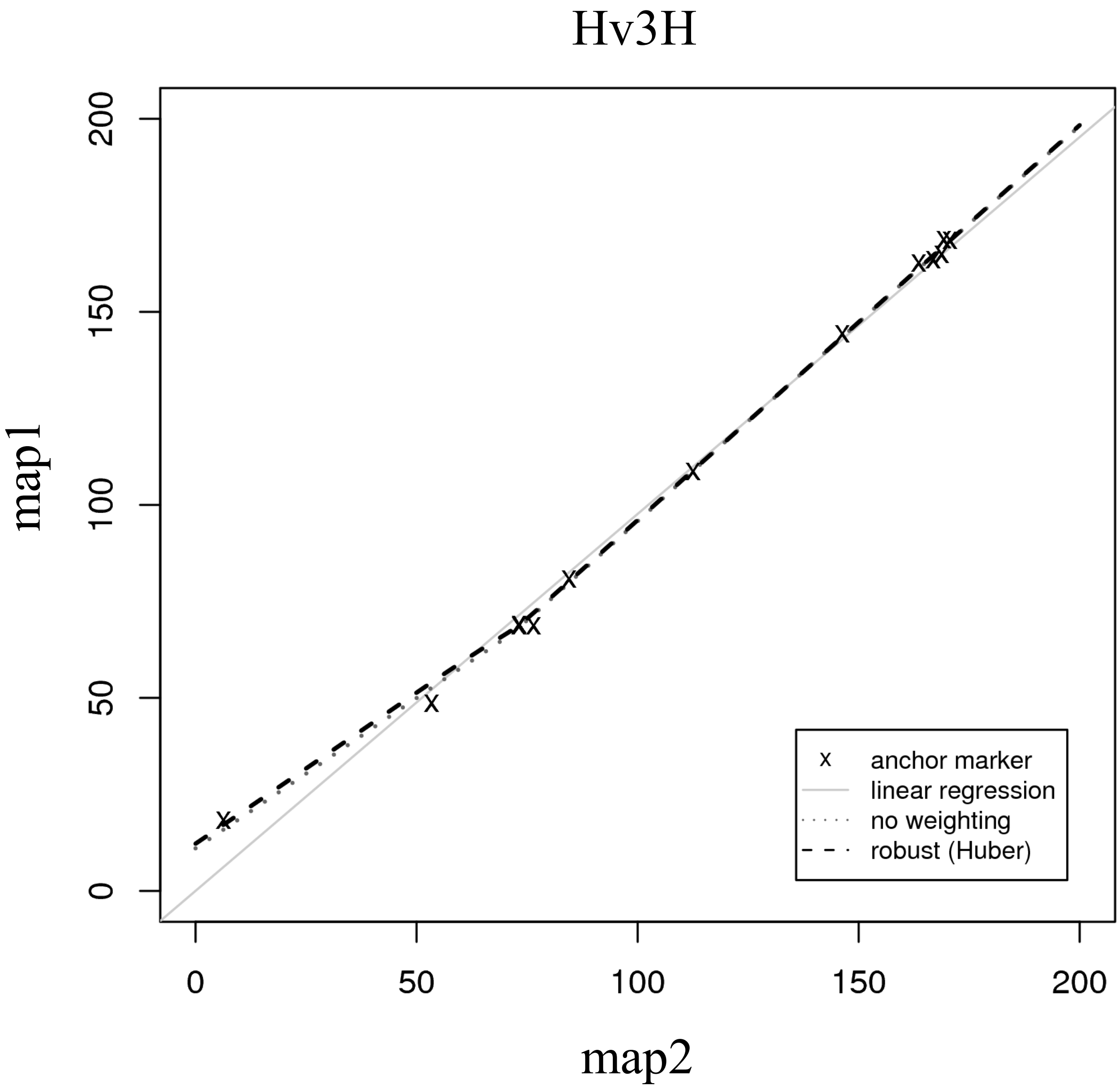

Figure S6 (continued)

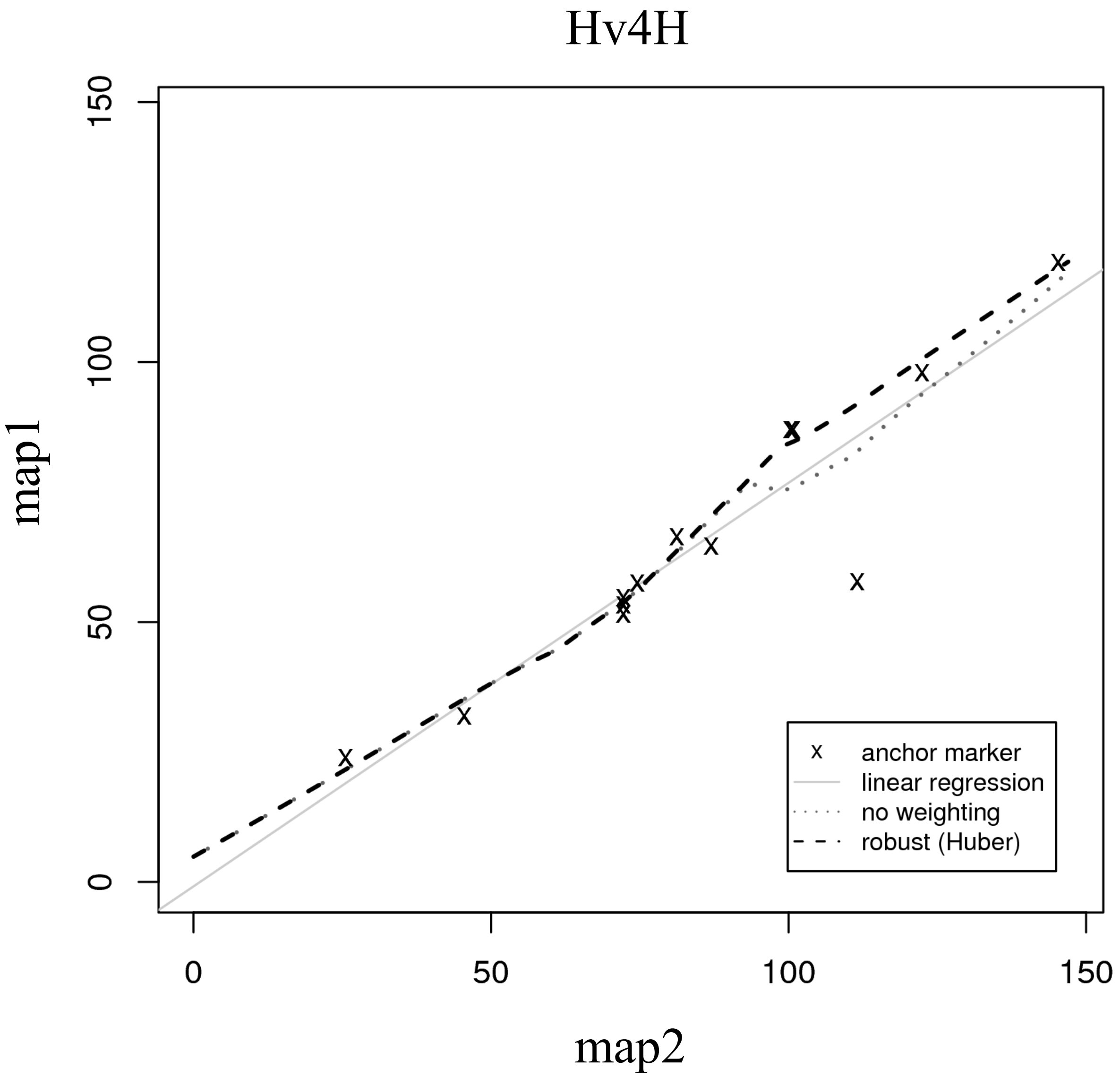

Figure S6 (continued)

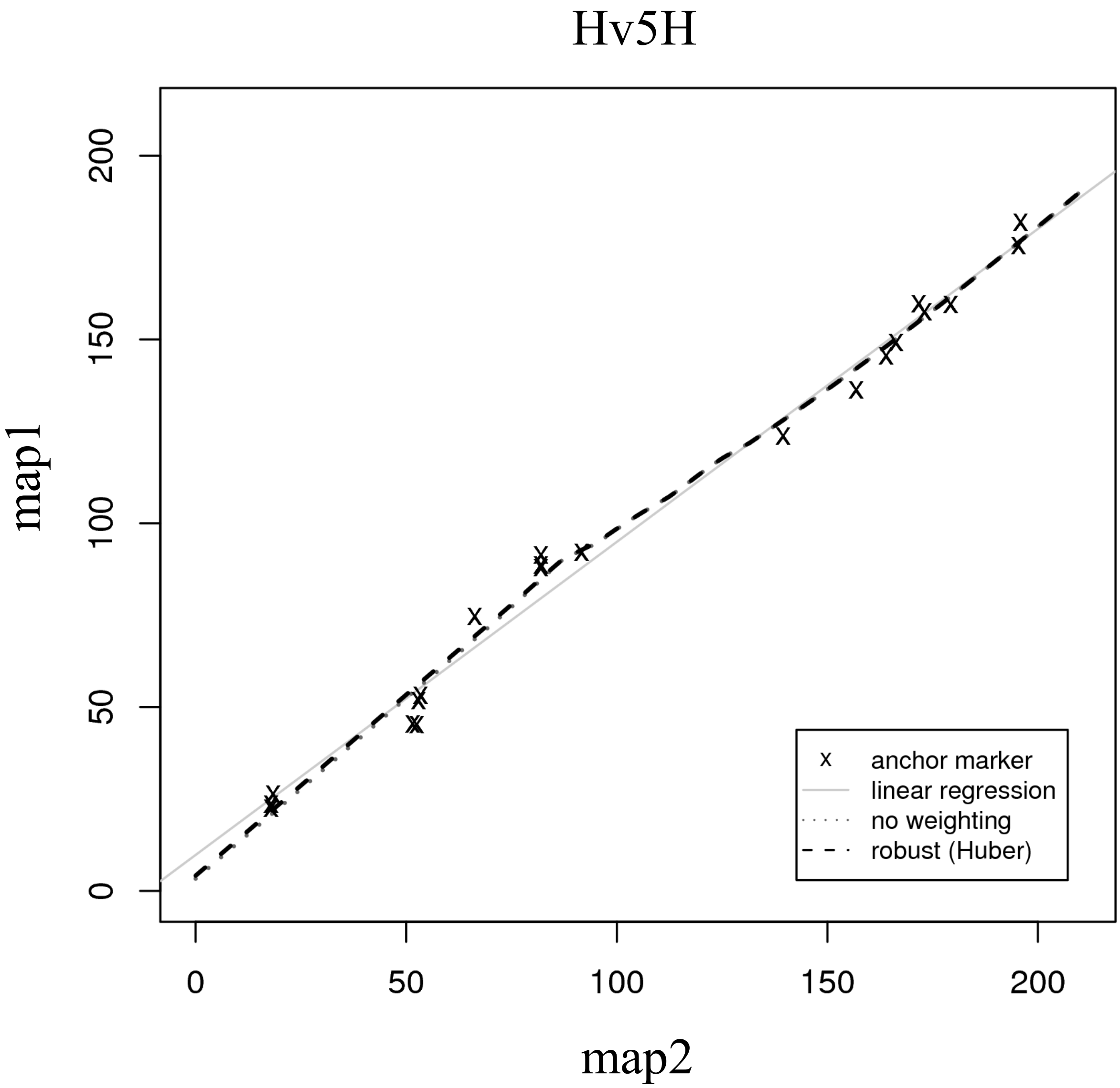

Figure S6 (continued)

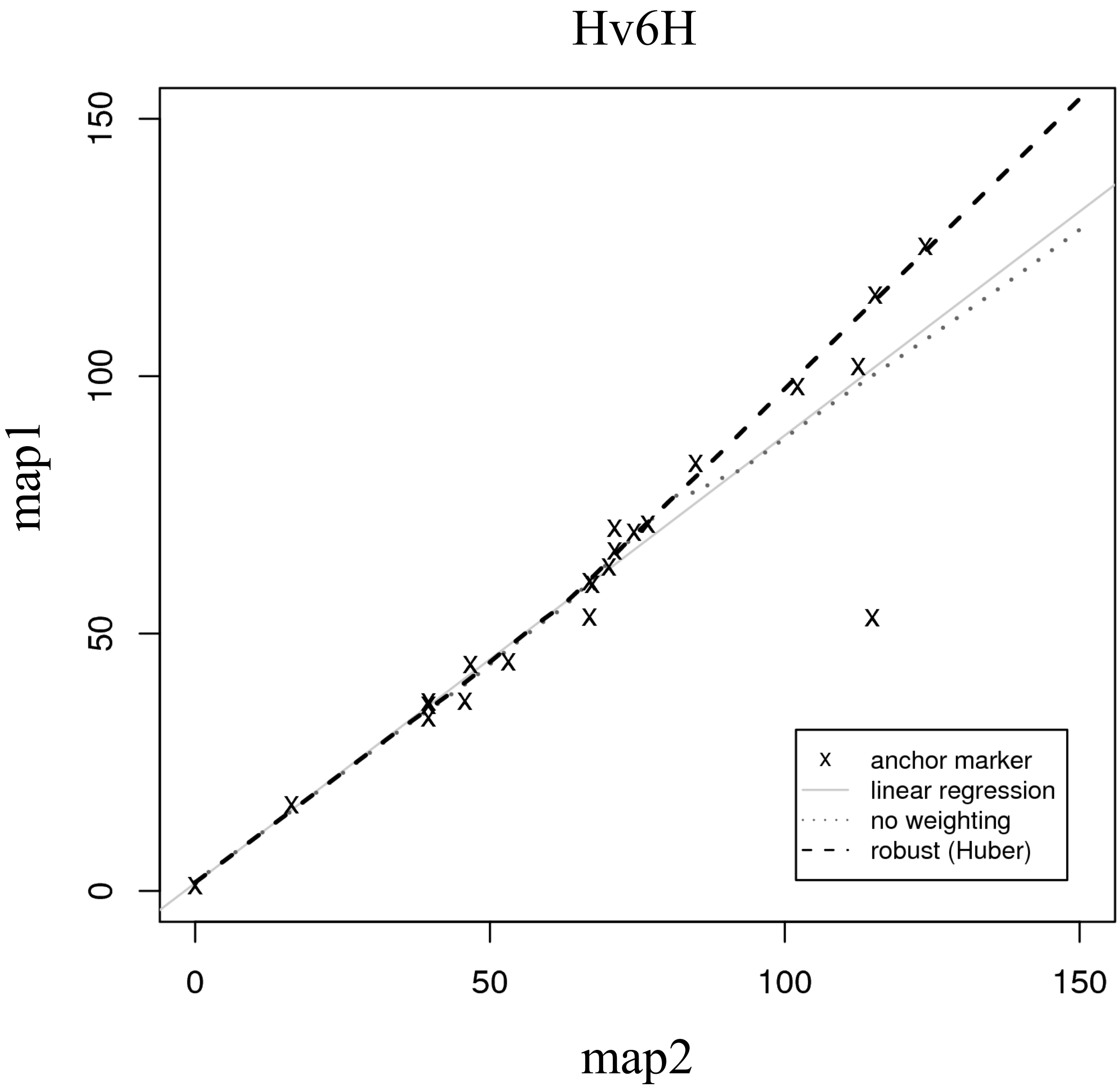

Figure S6 (continued)

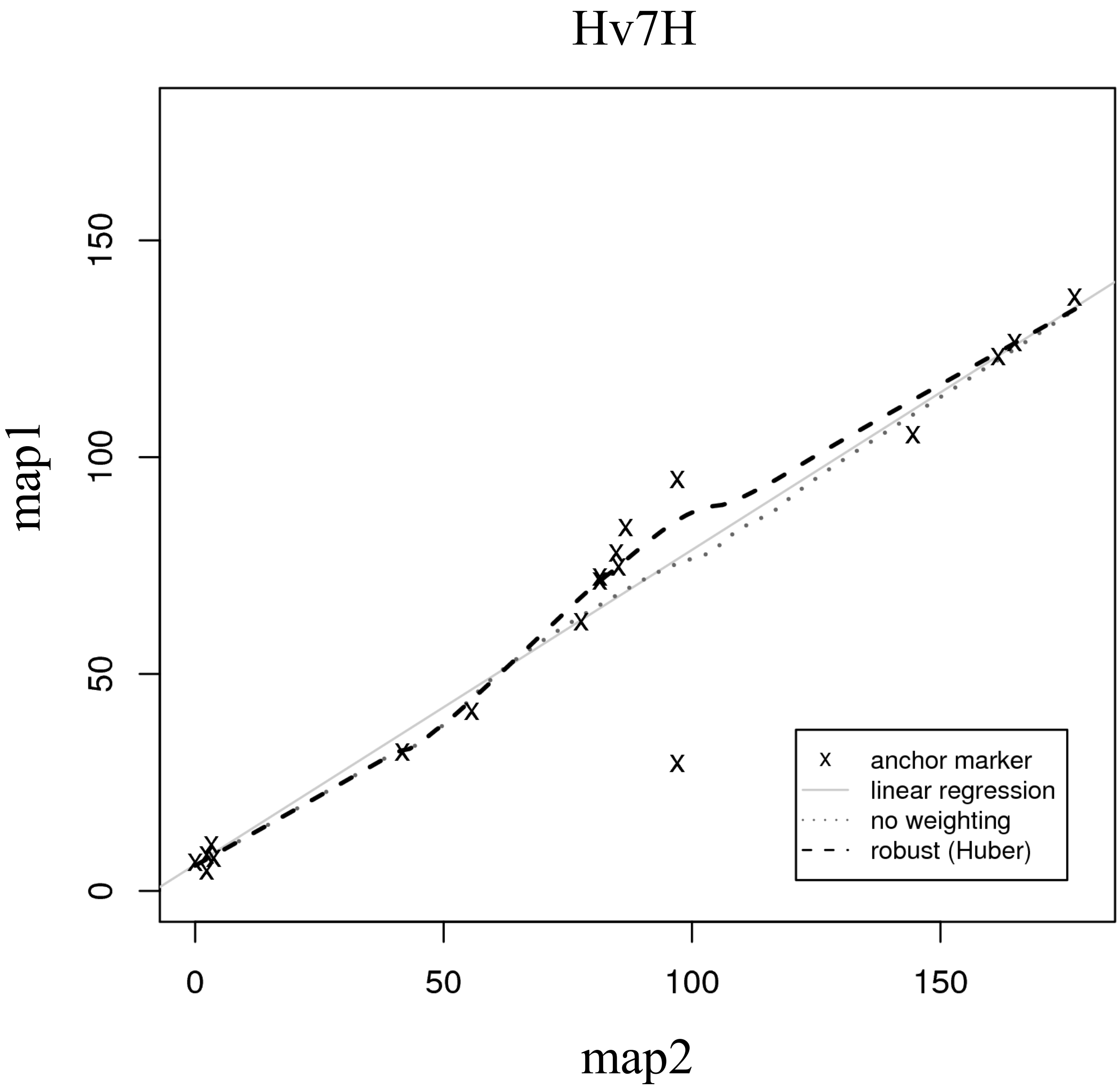

Figure S6 (continued)
